# Atroposelective Amination of Indoles via Chiral Center Induced Chiral Axis Formation

**DOI:** 10.3390/molecules27249008

**Published:** 2022-12-17

**Authors:** Yong Wang, Jingxue Yan, Yiqing Jiang, Zexuan Wei, Zhenlin Tu, Chao Dong, Tao Lu, Yadong Chen, Jie Feng

**Affiliations:** Key Laboratory of Natural Medicines, Department of Organic Chemistry, China Pharmaceutical University, Nanjing 210009, China

**Keywords:** central-to-axial, amination, atropisomers, indole skeleton, amino acid

## Abstract

The construction of an N–C chiral axis for N-aryl indole derivatives is meaningful as they widely exist in functionalized molecules. This work provides a novel method for this purpose via amination of amino acid derivatives at the C2 position of the indole and chiral center induced chiral axis formation. The protocol of this transformation is easily accessible, not requiring metal or an organic chiral catalyst, endowing this method with great potential in the construction of axis chiral *N*-aryl indoles.

## 1. Introduction

**Scheme 1:**
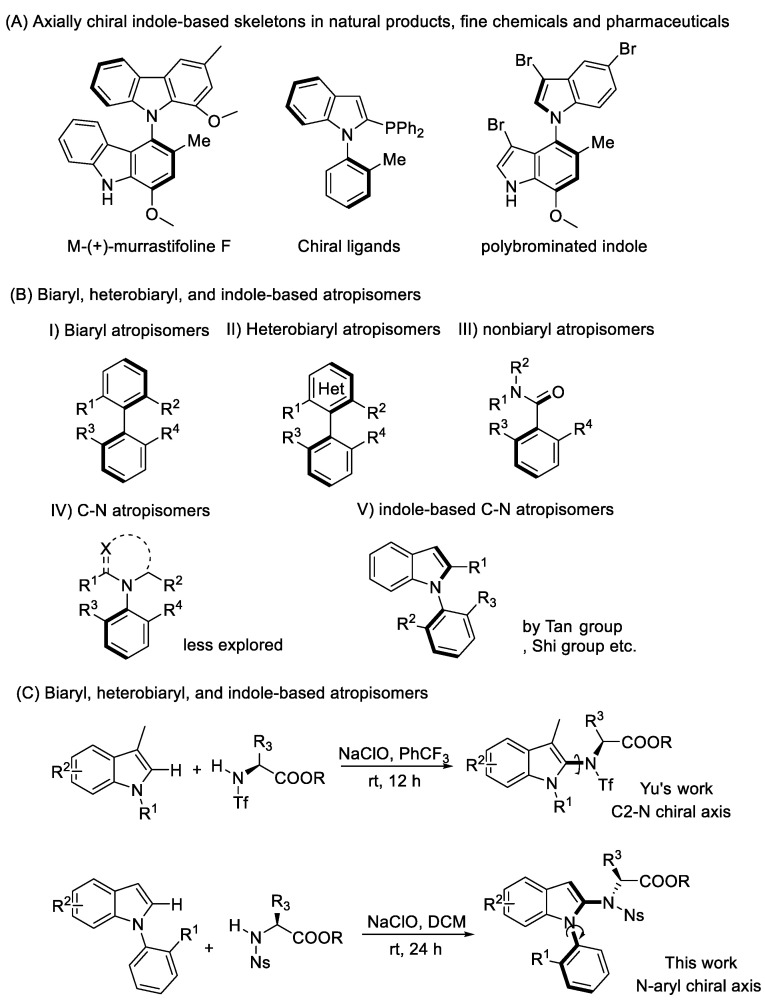
Functionalized axially chiral indole-based molecules and outline for indole axially chiral construction.

Atropisomers around the C–N chiral axis, which have a typical chiral axis connecting a substituted nitrogen and the aryl-related hindrance, are one of the most important classes of axially chiral compounds. For example, *Murrastifoline F* was isolated from murraya alkaloid, which possesses anti-HIV activity [1,2,3]. Moreover, such skeletons have been used as chiral organocatalysts and ligands in enantioselective reactions, due to their specific electronic properties among biaryls (Scheme 1a) [4,5,6].

Given the importance of C−N axially chiral biaryls in pharmaceuticals, organic synthesis, and materials, catalytic asymmetric syntheses of these C–N axially chiral compounds and their applications to asymmetric transformations are of current interest [7,8,9,10,11,12,13,14,15,16,17]. Additionally, it would be meaningful to introduce axial chirality into the indole skeleton, as the indole skeleton is widely apparent in many natural products and drug molecules. Catalytic asymmetric synthesis of indole-based heterobiaryl axially chiral compounds was disclosed by the Shi [18,19,20], Tan [21], Fu [22,23], and Yan [24] groups, etc. The chiral axis was normally built at the C2 or C3 position of the indole ring [25,26,27,28,29,30,31], while recent literature has reported the stereoselective synthesis of axially chiral indole derivatives possessing a sterically hindered phenyl group on the nitrogen atom [32,33,34,35]. Despite these promising achievements, asymmetric synthesis of indole-based atropisomers, especially with an N–C chiral axis, is still a synthetic challenge and remains largely unexplored (Scheme 1b).

This study tackles this synthetic defiance considering the asymmetric catalysis free and easy formation of indole-based heterobiaryl axially chiral compounds, in line with strategies such as chiral center induced chiral axis formation and the central-to-axial chirality conversion [36,37,38]. It is worth noting that the Yu’s group described a series of examples for atroposelective coupling of indoles at the C2 position with chiral amino acid-based sulfonamides via a central-to-axial chirality conversion strategy [39,40]. Encouraged by that, we rationalized that the construction of an N–C chiral axis for *N*-aryl indole derivatives possessing a sterically hindered *N*-phenyl group via amination at the C2 position of the indole and sequence chiral center induced chiral axis formation would represent a novel strategy for constructing indole-based N–C atropisomers (Scheme 1c).

## 2. Results and Discussion

To start this work, *N*-phenyl indole **1a** and tert-butyl ((4-nitrophenyl)sulfonyl)-L-valinate **2a** were used as the model substrate to test the feasibility for the C–N amination (Table 1). Product **3a** was afforded using an aqueous NaClO solution with **1a** in different solvents. The solvent was also crucial to both the yield and diastereoisomers (*dr*) value. The optimal solvent was found to be DCM in 65% yield and 3.5/1 *dr* value (entry 1–6). Only a trace product was observed in the protic solvent (entry 4). The yield loss was due to the formation of byproduct **4a** and oxidative byproduct **5a**. Interestingly, biaxial chirality was observed as the chlorine atom brings steric hindrance at the C3 position. In Yu’s work, excellent *dr* value was observed at the C–N axis chirality at the indole C2 position. Similar high *dr* was tracked for compound **4a** (entry 1).

In should be mentioned that the protecting group of valinate also significantly affected the yield and *dr* (Table 2). The *p*-nitrobenzene sulfonyl group (Ns) was favorable for this transformation (**entry 2**). Other sulfonyl protecting groups yielded the product **3a** in less than 5% yield. The benzyl protecting group (Bn) and tert-butoxycarbonyl group (Boc) were conductive to produce byproduct **5a.**

**Scheme 2:**
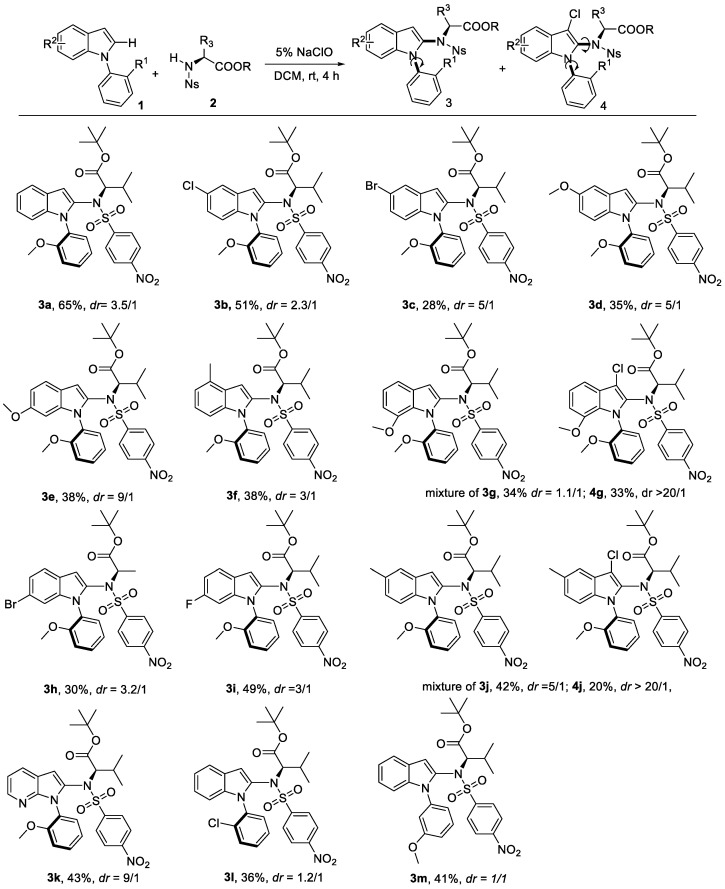
Substrates scope for various 1-(2-methoxyphenyl)-1H-indoles for construction of C–N axially chirality. reaction conditions: indoles (0.2 mmol), amino acid (0.6 mmol), 5% NaClO (0.6 mmol), DCM (4 mL), rt, 24 h.

In order to show the generality of this strategy, the couplings of various indoles with *N*-Ns protected amino acid derivatives were investigated under the optimal conditions. First, a series of substituted indole derivatives at different positions reacted with **2a** to give product **3a**–**3j** in moderate yield (Scheme 2, **3a**–**3h**). The electron-donating group bearing indoles at the C4-C5 position had lower yields but improved *dr* value (**3d**, **3e** vs. **3a**, **3b**, **3i**). For substrate **3j**, the C-3 chlorination product **4j** was produced at the same time. The *dr* value of **3j** was around 5/1, but its side product **4j** had excellent *dr* value. A similar *dr* value with the model substrate **3a** was obtained for C-6 substituted indoles (**3h**, **3i**). One example using the heterocyclic indole analogue with 1-(2-methoxyphenyl)-1H-pyrrolo[2,3-b]pyridine was also conducted with 43% yield and good *dr* value (**3k**). Next, we explored the variations at the *N*-aryl ring of the indole. A less bulky ortho-substituted substrate resulted in lower *dr* value (**3l**). In the case of substitutions at the meta position, 1:1 of two diastereomers were observed (**3m**).

**Scheme 3:**
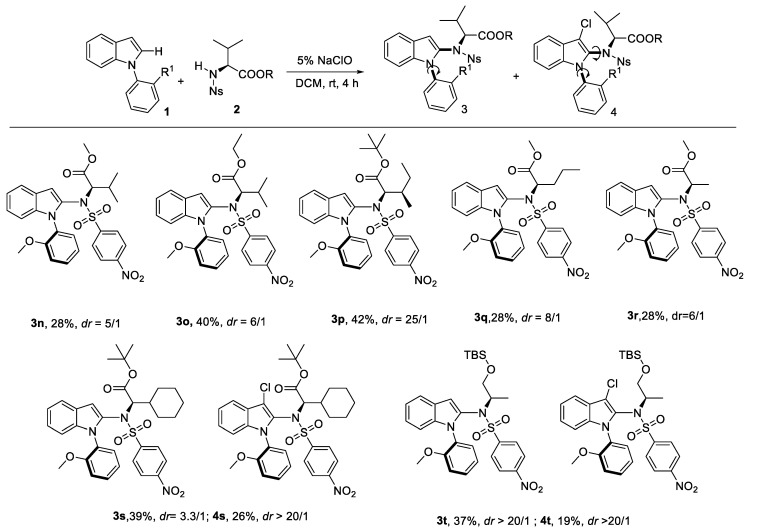
Substrates scope for various amino acid for construction of C–N axially chirality. reaction conditions: indoles (0.2 mmol), amino acid (0.6 mmol), 5% NaClO (0.6 mmol), DCM (4 mL), rt, 24 h.

After that, we continued to investigate the reactivity and stereoselectivity of the reactions of *N*-phenylindole (**1a**) with various amino acid derivatives (Scheme 3). Generally, the steric hindrance of amino acid derivatives had a positive influence on the stereoselectivity. To our delight, excellent *dr* value was shown with amino acid derivatives such as L-Isoleucine (**3p**), L-Norvaline (**3q**). But the yield was slightly decreased with bulker amino acid. As for the L-Cyclohexyl glycine derivative **3s** and 2-Amino-1-propanol derivative **3t**, byproducts **4s** and **4t** were tracked, respectively.

**Scheme 4:**
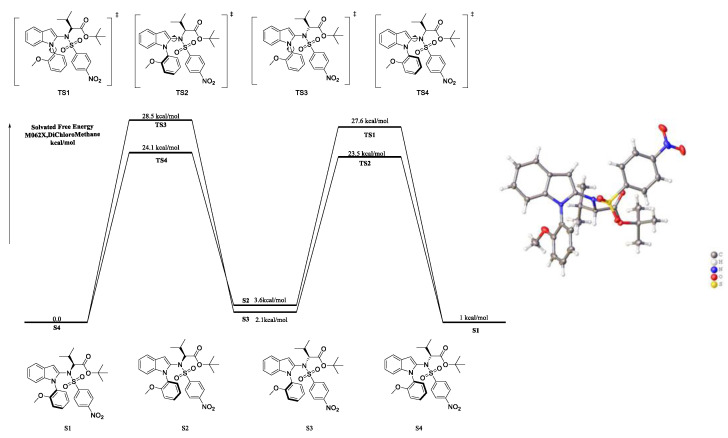
DFT calculations for rotate barrier for C–N axis and *N*-aryl axis and absolute configuration of **3a**.

The absolute configuration of axially chiral product **3a** was determined to be (Ra,S) by single-crystal X-ray diffraction analysis (Scheme 4) (CCDC 2222365 for the major diastereomer of 3a, see the Supporting Information for details). Thus, the absolute configurations of other axially chiral products were assigned to be (Ra,S) by analogy with **3a**. Next, we investigated the configurational stability and the rotational barrier of product **3a** and other isomers (S1–S4, Scheme 4) via DFT calculations (Scheme 4). It was discovered that the indole C2–N axis was unstable (the rotate barrier was 24.1 kcal/mol and 23.5 kcal/mol, respectively, for TS2 and TS4), which will rapidly disappear under room temperature. However, the N–C_aryl_ axis had good configurational stability due to the fact that the rotational barrier was high (28.5 kcal/mol for TS3, 27.6 kcal/mol for TS1). As the amino acid was optically pure, the diastereo ratio may come from the atropisomerism of an N–C_aryl_ axis.

In summary, we present here an atroposelective coupling of indoles with chiral amino acid-based sulfonamides mediated by hypo-halides through chiral center induced chiral axis formation strategy. The reaction delivers 2-amido-*N*-arylindoles with an N–C chiral axis in a moderate to good *dr* value. The substrates and reaction protocol of this transformation are easily accessible, not requiring chiral catalyst, endowing this method with great potential in the construction of axis chiral *N*-arylindoles.

## 3. Materials and Methods

Unless otherwise noted, all reactants or reagents including dry solvents were obtained from commercial suppliers and used as received. NaClO (Sodium hypochlorite solution reagent grade, available chlorine 4.00–4.99%) was purchased from Lingfeng reagent company, Shanghai, China. All the reactions were conducted using reaction tube (10 mL) under argon atmosphere. Analytical thin layer chromatography (TLC) was performed using Silica Gel 60 F25 plates. Column chromatograph was performed on silica gel 100~200 mesh. ^1^H and ^13^C NMR spectra were obtained in CDCl_3_ or DMSO using 300 MHz, 400 MHz Varian NMR spectrometer. Chemical shifts in ^1^H NMR spectra are reported in parts per million (ppm) on the δ scale from an internal standard of residual CDCl3 (7.26 ppm). Data are reported as follows: chemical shift, multiplicity (s = singlet, d = doublet, t = triplet, q = quartet, m = multiplet), integration, and coupling constant in Hertz (Hz). Chemical shifts in ^13^C NMR spectra are reported in ppm on the δ scale from the central peak of residual CDCl3 (77.16 ppm).

### 3.1. General Procedures for Indole C2 Amination with N-Ns Protected Amino Acid

A 10 mL round bottom flask was equipped with a rubber septum and magnetic stir bar and was charged with **1** (0.2 mmol), **2** (0.6 mmol), NaClO (0.6 mmol, 774 μL, 5% in water) in DCM (4 mL) at room temperature for 24 h. Upon completion of the reaction, the mixture was washed with saturated Na_2_CO_3_ aqueous solution (4 mL), water, and saturated brine (4 mL) in sequence. The organic layer was concentrated and purified via a flash column (PE/EA from 50/1 to 30/1).

### 3.2. Characterization Data for Compounds

*tert-butyl N-(1-(2-methoxyphenyl)-1H-indol-2-yl)-N-((4-nitrophenyl)sulfonyl)-D-valinate* **3a**. According to the general procedure, purification by flash chromatography, **3a** (75.4 mg, 65%, *dr* = 3.5:1) was obtained as a yellow solid from **1a** (0.2 mmol, 44.6 mg), **2a** (0.6 mmol, 215.0 mg), and NaClO (0.6 mmol, 774 μL, 5% in water). ^1^H NMR (300 MHz, CDCl_3_) δ 8.33 (d, *J* = 9.0 Hz, 2H × 0.78), 8.26 (d, *J* = 9.0 Hz, 2H × 0.22), 7.99 (d, *J =* 9.0 Hz, 2H × 0.78), 7.83 (d, *J* = 9.0 Hz, 2H × 0.22),7.56–7.37 (m, 3H), 7.14–6.89 (m, 4H), 6.57–6.54 (m, 1H), 6.01 (s, 1H × 0.22), 6.00 ((s, 1H × 0.78), 3.91 (d, *J =* 9.0 Hz, 1H × 0.78)), 3.56 (d, *J =* 9.0 Hz, 1H × 0.22), 3.50 (s, 3H × 0.78), 3.47 (s, 1H × 0.22), 1.55–1.41 (m, 1H × 0.78), 1.05 (s, 9H × 0.22), 0.94 (s, 9H × 0.78), 0.54 (d, *J* = 6.6 Hz, 3H × 0.22), 0.47 (d, *J* = 6.5 Hz, 3H × 0.78), 0.14 (d, *J* = 6.7 Hz, 3H × 0.78). ^13^C NMR (75 MHz, Chloroform-*d*) δ 167.4, 156.5, 150.5, 145.0, 137.2, 132.7, 130.6, 125.0, 124.8, 123.8, 123.5, 121.3, 121.0, 120.5, 111.7, 111.4, 104.6, 82.3, 72.1, 55.3, 29.1, 27.9, 19.6, 19.5. HRMS Calcd. For C_30_H_33_N_3_NaO_7_S [M+Na]^+^: 602.1931; found: 602.1920.

*tert-butyl N-(5-chloro-1-(2-methoxyphenyl)-1H-indol-2-yl)-N-((4-nitrophenyl)sulfonyl)-D-valinate* **3b**. According to the general procedure, purification by flash chromatography, **3b** (66.2 mg, 51%, *dr* = 2.3:1) was obtained as a yellow solid from **1b** (0.2 mmol, 51.5 mg), **2a** (0.6 mmol, 215.0 mg), and NaClO (0.6 mmol, 774 μL, 5% in water). ^1^H NMR (300 MHz, Chloroform-*d*) δ 8.51 (d, *J =* 6.0 Hz, 2H × 0.70), 8.42 (d, *J =* 6.0 Hz, 2H × 0.30), 8.23 (d, *J* = 6.0 Hz, 2H × 0.7), 8.16–8.12 (m, 1H), 8.04 (d, *J* = 6.0 Hz, 2H × 0.3), 7.64–7.59 (m, 2H), 7.46–7.38 (m, 1H), 7.32–7.16 (m, 3H), 6.88 (d, *J* = 8.7 Hz, 1H), 6.19 (s, 1H × 0.32), 6.10 (1H × 0.76), 4.33–4.17 (m, 1H), 3.82–3.79 (m, 3H), 1.82–1.79 (m, 1H × 0.70), 1.43 (s, 9H × 0.30), 1.31 (s, 9H × 0.70), 1.13–1.09 (m, 3H × 0.30), 0.90 (d, *J =* 9.0 Hz, 3H × 0.30), 0.81 (d, *J =* 9.0 Hz, 3H × 0.3), 0.55 (d, *J =* 9.0 Hz, 3H × 0.70). ^13^C NMR (75 MHz, Chloroform-*d*) δ 167.3, 156.2, 150.4, 144.8, 135.2, 132.4, 132.1, 131.0, 130.8, 130.5, 128.7, 126.2, 126.1, 125.7, 124.3, 124.2, 123.9, 123.8, 123.4, 121.4, 120.3, 120.2, 113.0, 111.5, 104.1, 82.4, 77.4, 71.8, 66.0, 55.5, 55.3, 30.4, 29.2, 28.1, 27.9, 27.9, 27.8, 19.7, 19.4, 19.2. HRMS Calcd. For C_30_H_32_ClN_3_NaO_7_S [M+Na]^+^: 636.1542; found: 636.1530.

*tert-butyl N-(5-bromo-1-(2-methoxyphenyl)-1H-indol-2-yl)-N-((4-nitrophenyl)sulfonyl)-D-valinate* **3c** According to the general procedure, purification by flash chromatography, **3c** (3.88 mg, 28%, *dr* =5:1) was obtained as a yellow solid from **1c** (0.2 mmol, 60.4 mg), **2a** (0.6 mmol, 215.0 mg), and NaClO (0.6 mmol, 774 μL, 5% in water). ^1^H NMR (300 MHz, Chloroform-*d*) δ 8.39 (d, *J =* 9.0 Hz, 2H × 0.83), 8.34 (d, *J =* 9.0 Hz, 2H × 0.17), 8.30 (d, *J* = 9.0 Hz, 2H × 0.17) 8.12–8.00 (m, 2H × 0.83), 8.02 (d, *J =* 9.0 Hz, 2H × 0.17), 7.91 (d, *J =* 6.0 Hz, 2H), 7.86 (d, *J =* 3.0 Hz, 1H × 0.83), 7.66 (d, J = 3.0 Hz, 1H × 0.17), 7.53–7.47 (m, 1H), 7.42 (dd, *J* = 9.0 Hz, 3.0 Hz, 1H × 0.83), 7.32 (dd, J = 9.0 Hz, 3.0 Hz, 1H × 0.17), 7.19–7.14 (m, 1H), 7.07–7.04 (m, 1H), 6.73 (d, *J* = 8.7 Hz, 1H × 0.17), 6.62 (d, *J* = 8.7 Hz, 1H × 0.83), 6.05 (s, 1H × 0.17), 5.98 (s, 1H × 0.83), 4.20 (d, *J* = 9.2 Hz, 1H × 0.83), 4.07 (d, *J* = 9.2 Hz, 1H × 0.17), 3.70 (s, 3H × 0.17), 3.67 (s, 3H × 0.83), 2.15–1.58 (m, 1H), 1.31 (s, 9H × 0.17), 1.20 (s, 9H × 0.83), 1.00–0.42 (m, 6H). Crude ^13^C NMR (101 MHz, Chloroform-*d*) δ 171.2, 168.9, 167.3, 156.2, 150.4, 150.2, 144.8, 136.0, 132.4, 131.9, 131.9, 131.7, 131.0, 130.9, 130.5, 129.6, 129.6, 128.7, 127.3, 126.5, 124.2, 124.2, 123.8, 123.4, 123.3, 121.4, 113.8, 113.4, 111.5, 104.0, 103.7, 84.1, 84.0, 82.4, 82.3, 71.8, 66.0, 60.5, 55.5, 55.3, 53.6, 30.4, 29.2, 28.1, 28.0, 27.9, 27.9, 19.7, 19.4, 19.4, 19.2, 14.3. HRMS of **3c** Calcd. For C_30_H_32_BrN_3_NaO_7_S [M+Na]^+^: 680.1037; found: 680.1032.

*tert-butyl N-(5-methoxy-1-(2-methoxyphenyl)-1H-indol-2-yl)-N-((4-nitrophenyl)sulfonyl)-D-valinate***3d**. According to the general procedure, purification by flash chromatography, **3d** (42.7 mg, 35%, *dr* =5:1) was obtained as a yellow solid from **1d** (0.2 mmol, 50.7 mg), **2a** (0.6 mmol, 215.0 mg), and NaClO (0.6 mmol, 774 μL, 5% in water). ^1^H NMR (300 MHz, Chloroform-*d*) δ 8.39 (d, *J =* 9.0 Hz, 2H × 0.83), 8.30 (d, *J =* 9.0 Hz, 2H × 0.17), 8.14 (d, *J* = 9.0 Hz, 2H × 0.83), 7.92 (d, *J =* 9.0 Hz, 2H × 0.17), 7.56–7.45 (m, 2H), 7.36 (t, *J* = 2.2 Hz, 1H), 7.18–7.11 (m, 1H), 7.04 (dd, *J* = 8.3, 1.3 Hz, 1H), 6.95 (d, *J* = 2.4 Hz, 1H), 6.86–6.83 (m, 1H), 6.74 (d, *J* = 8.9 Hz, 1H), 6.03 (s, 1H × 0.17), 5.92 (s, 1H × 0.8), 4.21 (d, *J* = 9.4 Hz, 1H), 3.84 (s, 3H), 3.68 (s, 3H), 1.74–1.71 (m, 1H), 1.29 (s, 9H), 0.97 (d, J = 6.0 Hz, 3H × 0.17), 0.79 (d, *J* = 6.0 Hz, 3H × 0.17), 0.70 (d, J = 6.0 Hz, 3H × 0.83), 0.43 (d, J = 6.0 Hz, 3H × 0.83). ^13^C NMR (101 MHz, Chloroform-*d*) δ 167.4, 157.5, 156.4, 150.4, 145.1, 137.8, 132.7, 130.6, 130.6, 129.8, 124.9, 123.7, 121.7, 121.4, 119.1, 111.5, 110.8, 104.7, 94.7, 82.2, 72.1, 55.7, 55.3, 29.1, 28.0, 27.9, 19.6, 19.5. HRMS Calcd. For C_31_H_35_N_3_NaO_8_S [M+Na]^+^: 632.2037; found: 632.2037.

*tert-butyl N-(6-methoxy-1-(2-methoxyphenyl)-1H-indol-2-yl)-N-((4-nitrophenyl)sulfonyl)-D-valinate***3e**. According to the general procedure, purification by flash chromatography, **3e** (46.3 mg, 38%, *dr* =9:1) was obtained as a yellow solid from **1e** (0.2 mmol, 50.7 mg), **2a** (0.6 mmol, 215.0 mg), and NaClO (0.6 mmol, 774 μL, 5% in water). ^1^H NMR (300 MHz, Chloroform-*d*) δ 8.40 (d, *J =* 9.0 Hz, 2H × 0.90), 8.31(d, *J =* 9.0 Hz, 2H × 0.1) 7.95 (d, *J =* 6.0 Hz, 1H), 7.59–7.49 (m, 1H), 7.40 (d, *J* = 8.6 Hz, 1H × 0.9), 7.34 (d, *J* = 8.6 Hz, 1H × 0.1), 7.20 (d, *J* = 1.3 Hz, 1H), 7.07 (dd, *J* = 8.4, 1.3 Hz, 1H), 6.81 (dd, *J* = 8.7, 2.3 Hz, 1H), 6.29 (d, *J* = 2.3 Hz, 1H), 6.03 (s, 1H × 0.1), 5.93 (d, *J =* 0.72 Hz, 1H × 0.90), 4.22 (d, *J* = 9.4 Hz, 1H × 0.9), 3.97 (d, *J* = 9.4 Hz, 1H × 0.1), 3.74 (s, 3H), 3.71 (s, 3H), 1.77–1.70 (m, 1H), 1.33–1.21 (m, 9H), 0.84–0.43 (m, 6H). ^13^C NMR (101 MHz, CDCl_3_) δ 167.45, 157.55, 156.41, 150.35, 145.08, 137.84, 132.70, 130.60, 130.56, 129.82, 128.77, 124.85, 124.31, 123.73, 121.78, 121.71, 121.38, 119.12, 111.47, 110.84, 104.72, 94.68, 82.18, 77.48, 77.16, 76.84, 72.09, 55.74, 55.30, 29.07, 27.99, 27.87, 19.64, 19.49, 19.21. HRMS Calcd. For C_31_H_35_N_3_NaO_8_S [M+Na]^+^: 632.2037; found: 632.2037.

*tert-butyl N-(1-(2-methoxyphenyl)-4-methyl-1H-indol-2-yl)-N-((4-nitrophenyl)sulfonyl)-D-valinate***3f**. According to the general procedure, purification by flash chromatography, **3f** (45.1 mg, 38%, *dr* =3:1) was obtained as a yellow solid from **1f** (0.2 mmol, 47.5 mg), **2a** (0.6 mmol, 215.0 mg), and NaClO (0.6 mmol, 774 μL, 5% in water). ^1^H NMR (300 MHz, Chloroform-*d*) δ 8.57–8.48 (m, 2H), 8.32–8.05 (m, 3H), 7.65 (d, *J* = 7.8 Hz, 1H), 7.36–7.21 (m, 3H), 7.10–7.08 (m, 1H), 6.85 (d, *J* = 8.3 Hz, 1H), 6.30 (s, 1H × 0.25), 6.17 (s, 1H × 0.75), 4.34 (d, *J* = 9.6 Hz, 1H), 3.85 (s, 3H), 2.57 (s, 3H), 1.97–1.87 (m, 1H), 1.49 (s, 9H × 0.25), 1.38 (s, 9H × 0.75), 1.17 (d, *J =* 6.0 Hz, 3H × 0.25), 0.97 (d, *J =* 6.0 Hz, 3H × 0.25), 0.90 (d, *J =* 6.0 Hz, 3H × 0.75), 0.61 (d, *J =* 6.0 Hz, 3H × 0.75). ^13^C NMR (75 MHz, Chloroform-*d*) δ 167.4, 156.5, 150.4, 145.0, 136.8, 132.6, 130.8, 130.7, 130.6, 130.4, 130.3, 125.0, 124.9, 123.6, 121.3, 120.7, 120.6, 111.4, 109.4, 103.1, 82.2, 72.2, 58.6, 55.3, 29.0, 28.0, 27.9, 19.6, 19.5, 18.7, 18.6. HRMS Calcd. For C_31_H_35_N_3_NaO_7_S [M+Na]^+^: 616.2088; found: 616.2074.

*tert-butyl N-(7-methoxy-1-(2-methoxyphenyl)-1H-indol-2-yl)-N-((4-nitrophenyl)sulfonyl)-D-valinate* **3g**. According to the general procedure, purification by flash chromatography, **3g** (37.4 mg, 34 %, *dr* = 1.1:1) was obtained as a yellow solid from **1g** (0.2 mmol, 50.6 mg), **2a** (0.6 mmol, 215.0 mg), and NaClO (0.6 mmol, 774 μL, 5% in water). Crude ^1^H NMR of **3g**. ^1^H NMR (300 MHz, Chloroform-*d*) δ 8.40 (d, *J* = 8.8 Hz, 2H × 0.52), 8.30 (d, *J* = 8.5 Hz, 2H × 0.48), 8.14–7.94 (m, 2H), 7.43–7.34 (m, 2H), 7.19–6.89 (m, 5H), 6.66 (d, *J* = 7.8 Hz, 1H), 6.15 (s, 1H × 0.48), 6.01 (s, 1H × 0.52), 4.26–4.07 (m, 1H), 3.79 (s, 3H × 0.48), 3.79 (s, 3H × 0.52), 3.50 (s, 3H × 0.52), 3.50 (s, 3H × 0.48), 1.75–1.60 (m, 1H), 1.35 (s, 9H × 0.48), 1.23 (s, 9H × 0.52), 0.95–0.41 (m, 6H). Crude ^13^C NMR (75 MHz, Chloroform-*d*) δ 167.4, 157.3, 155.8, 150.2, 150.0, 147.6, 145.7, 144.9, 131.4, 131.0, 130.6, 130.3, 130.1, 129.7, 129.3, 127.5, 127.1, 126.8, 124.7, 124.6, 123.6, 123.1, 120.6, 120.5, 120.3, 120.1, 117.2, 115.9, 113.9, 111.4, 110.1, 105.2, 105.1, 104.9, 82.1, 71.9, 56.4, 55.9, 55.1, 53.5, 29.0, 27.9, 27.8, 19.6, 19.4, 1.1. HRMS of **3g** Calcd. For C_31_H_35_N_3_NaO_8_S [M+Na]^+^: 632.2037; found: 632.2024.

*tert-butyl N-(6-bromo-1-(2-methoxyphenyl)-1H-indol-2-yl)-N-((4-nitrophenyl)sulfonyl)-D-alaninate* **3h**. According to the general procedure, purification by flash chromatography, **3h** (37.8 mg, 30%, *dr* =3.2:1) was obtained as a yellow solid from **1h** (0.2 mmol, 60.4 mg), **2a** (0.6 mmol, 215.0 mg), and NaClO (0.6 mmol, 774 μL, 5% in water). ^1^H NMR (300 MHz, Chloroform-*d*) δ 8.38 (d, *J =* 9.0 Hz, 2H × 0.76), 8.30 (d, *J =* 9.0 Hz, 2H × 0.24), 8.10 (d, *J =* 9.0 Hz, 2H × 0.76), 8.03–8.78 (m, 1H × 1.48), 7.58–7.46 (m, 1H), 7.40–7.36 (m, 1H), 7.28–7.21 (m, 1H), 7.21–6.99 (m, 3H), 6.09 (s, 1H × 0.23), 6.00 (s, 1H), 4.21–4.05 (m, 1H), 3.72 (s, 3H × 0.23), 3.69 (s, 3H × 0.77), 1.30 (s, 9H × 0.23), 1.19 (s, 9H × 0.74), 1.01–0.41(m, 6H). ^13^C NMR (101 MHz, Chloroform-*d*) δ 167.4, 156.3, 150.4, 144.8, 138.0, 132.5, 131.5, 131.0, 130.6, 129.4, 128.8, 124.2, 123.8, 122.6, 121.4, 120.6, 114.6, 111.6, 104.8, 87.9, 82.4, 71.9, 55.4, 29.2, 28.0, 27.9, 19.7, 19.4. HRMS of **3h** Calcd. For C_30_H_32_BrN_3_NaO_7_S [M+Na]^+^: 680.1037; found: 680.1026.

*tert-butyl N-(6-fluoro-1-(2-methoxyphenyl)-1H-indol-2-yl)-N-((4-nitrophenyl)sulfonyl)-D-valinate***3i**. According to the general procedure, purification by flash chromatography, **3i** (58.6 mg, 49%, *dr* =2.9:1) was obtained as a yellow solid from **1i** (0.2 mmol, 48.3 mg), **2a** (0.6 mmol, 215.0 mg), and NaClO (0.6 mmol, 774 μL, 5% in water). HPLC retention time and peak area %: 4.54 min of 71.39%, and 5.05 min of 24.63%. ^1^H NMR (300 MHz, Chloroform-*d*) δ 8.39 (d, *J* = 9.3 Hz, 2H × 0.26), 8.34 (d, *J* = 9.3 Hz, 2H × 0.26), 8.12 (d, *J* = 8.9 Hz, 2H × 0.74), 8.02 (d, *J* = 8.9 Hz, 2H × 0.74), 7.92 (d, *J* = 7.6 Hz, 1H), 7.53–7.40 (m, 2H), 7.17 (m, 1H), 7.06 (d, *J* = 9.7 Hz, 1H), 6.89 (m, 1H), 6.52 (dd, *J* = 9.8, 2.3 Hz, 1H), 6.10 (s, 1H × 0.26), 6.00 (s, 1H × 0.74), 4.20 (d, *J* = 9.4 Hz, 1H × 0.74), 4.07 (d, *J* = 9.4 Hz, 1H × 0.76), 3.69 (s, 3H × 0.74), 2.93 (s, 1H × 0.26), 2.15–2.05 (m, 1H × 0.26), 1.74–1.66 (m, 1H × 0.74), 1.26 (s, 9H × 0.24), 1.19 (s, 9H × 0.76), 0.99 (d, *J* = 3.0 Hz, 1H), 0.78 (d, *J* = 6.6 Hz, 3H × 0.24), 0.69 (d, *J* = 6.6 Hz, 3H × 0.76), 0.42 (d, *J* = 6.6 Hz, 3H × 0.74). ^13^C NMR (75 MHz, Chloroform-*d*) δ 168.9, 167.4, 162.4, 159.3, 156.2, 150.4, 150.0, 144.9, 137.2, 137.0, 132.4, 131.3, 131.0, 130.8, 130.5, 128.7, 124.4, 124.3, 123.8, 122.1, 121.9, 121.4, 121.3, 111.5, 109.7, 109.4, 104.7, 98.1, 97.8, 82.3, 77.4, 71.9, 66.0, 55.3, 30.4, 29.1, 27.9, 27.8, 19.6, 19.2. HRMS Calcd. For C_30_H_32_FN_3_NaO_7_S [M+Na]^+^: 620.1837; found: 620.1826.

*tert-butyl N-(1-(2-methoxyphenyl)-5-methyl-1H-indol-2-yl)-N-((4-nitrophenyl)sulfonyl)-D-valinate***3j**. According to the general procedure, purification by flash chromatography, **3j** (49.9 mg, 42%, *dr* =5:1) and **4j** (23.7 mg, 20%, *dr* > 20:1) was obtained as a yellow solid from **1j** (0.2 mmol, 47.5 mg), **2a** (0.6 mmol, 215.0 mg), and NaClO (0.6 mmol, 774 μL, 5% in water). HPLC retention time and peak area %: 3.48 min of 2.05%, 3.82 min of 25.50%, 4.18 min of 4.11% and 4.83 min of 62.76%. Crude ^1^H NMR of **3j** and **4j** (300 MHz, Chloroform-*d*) δ 8.38–8.26 (m, 2.5H), 8.13–7.89 (m, 2.5H), 7.50–7.43 (m, 2H), 7.35–7.28 (m, 2H), 7.18–6.99 (m, 5H), 6.74 (d, *J* = 8.4 Hz, 1H), 6.42 (d, *J* = 8.4 Hz, 0.5 H), 6.01 (s, 1H × 0.16), 5.90 (s, 1H × 0.83), 4.21–4.05 (m, 1.3H), 3.79–3.67 (m, 4H), 2.42 (s, 3H), 2.38 (s, 1H), 1.78–1.66 (m, 1H), 1.32–1.20 (m, 12H), 1.01–0.42 (m, 8H). ^13^C NMR (75 MHz, CDCl_3_) δ 167.42, 156.46, 155.54, 150.32, 145.05, 139.11, 135.36, 134.16, 132.63, 132.23, 130.99, 130.80, 130.63, 130.47, 129.70, 129.44, 128.75, 125.36, 125.21, 125.00, 124.95, 124.27, 123.72, 121.31, 121.28, 120.45, 112.73, 111.40, 110.44, 104.18, 82.20, 72.01, 56.01, 55.30, 29.08, 27.99, 27.95, 27.88, 21.54, 21.16, 19.67, 19.47. HRMS Calcd. For C_31_H_35_N_3_NaO_7_S [M+Na]^+^: 616.2088; found: 616.2075.

*tert-butyl N-(1-(2-methoxyphenyl)-1H-pyrrolo[2,3-b]pyridin-2-yl)-N-((4-nitrophenyl)sulfonyl)-D-valinate***3k**. According to the general procedure, purification by flash chromatography, **3k** (49.9 mg, 43 %, *dr* = 9:1) was obtained as a yellow solid from **1k** (0.2 mmol, 44.9 mg), **2a** (0.6 mmol, 215.0 mg), and NaClO (0.6 mmol, 774 μL, 5% in water). ^1^H NMR (300 MHz, Chloroform-*d*) δ 8.40–8.38 (m, 3H × 0.9), 8.35–8.28 (m, 3H × 0.1), 8.10–8.05 (d, *J* = 7.9 Hz, 2H × 0.9), 8.03–7,85 (m, 2H × 0.1),7.53–7.47 (m, 1H), 7. 20–7.05 (m, 3H), 6.20 (s, 1H × 0.1), 6.09 (s, 1H × 0.9), 4.21 (d, *J* = 9.2 Hz, 1H), 3.66 (s, 3H × 0.1), 3.66 (s, 3H × 0.9), 1.79–1.69 (m, 1H), 1.32 (s, 9H × 0.1), 1.22 (s, 9H × 0.9), 0.79–0.44 (m, 6H). ^13^C NMR (75 MHz, Chloroform-*d*) δ 167.6, 156.6, 150.4, 147.5, 145.6, 144.8, 132.3, 132.1, 131.1, 130.5, 129.5, 123.9, 123.9, 121.3, 118.1, 117.1, 112.0, 103.4, 82.4, 71.6, 55.7, 29.2, 28.0, 27.9, 19.8, 19.4. HRMS Calcd. For C_29_H_33_N_4_O_7_S [M+H]^+^:581.2064; found: 581.2050.

*tert-butyl N-(1-(2-chlorophenyl)-1H-indol-2-yl)-N-((4-nitrophenyl)sulfonyl)-D-valinate***3l**. According to the general procedure, purification by flash chromatography, **3l** (42.0 mg, 36%, *dr* = 1.2:1) was obtained as a yellow solid from **1l** (0.2 mmol, 45.5 mg), **2a** (0.6 mmol, 215.0 mg), and NaClO (0.6 mmol, 774 μL, 5% in water). ^1^H NMR (300 MHz, Chloroform-*d*) δ 8.43–8.40 (m, 2H × 0.55), 8.40–8.30 (m, 2H × 0.45), 8.17–8.11 (m, 3H), 7.62–7.48 (m, 4H), 7.22–7.12 (m, 1H), 6.90–6.84 (m, 1H), 6.09 (s, 1H × 0.45), 5.91 (s, 1H × 0.55), 4.23 (m, 1H), 0.89–0.79 (m, 1H), 1.31 (s, 9H × 0.45), 1.15 (s, 9H × 0.55), 0.83–0.75 (m, 3H), 0.83–0.52 (m, 3H). ^13^C NMR (75 MHz, CDCl_3_) δ 167.2, 150.5, 150.2, 144.6, 136.1, 134.0, 133.4, 130.8, 130.8, 130.5, 130.5, 128.3, 125.2, 124.9, 124.1, 124.1, 123.8, 123.2, 121.3, 121.2, 121.1, 121.0, 111.8, 105.2, 82.6, 73.1, 53.6, 29.8, 29.1, 28.0, 27.8, 19.8, 18.7. HRMS Calcd. For C_29_H_30_ClN_3_O_6_S [M+H]^+^:606.1436; found: 606.1434.

*tert-butyl N-(1-(3-methoxyphenyl)-1H-indol-2-yl)-N-((4-nitrophenyl)sulfonyl)-D-valinate***3m**. According to the general procedure, purification by flash chromatography, **3m** (47.5 mg, 41%) was obtained as a yellow solid from **1m** (0.2 mmol, 44.7 mg), **2a** (0.6 mmol, 215.0 mg), and NaClO (0.6 mmol, 774 μL, 5% in water). ^1^H NMR (300 MHz, Chloroform-*d*) δ 8.39 (d, *J* = 8.6 Hz, 2H), 8.12 (d, *J* = 8.5 Hz, 2H), 7.52 (d, *J* = 7.9 Hz, 1H), 7.45 (d, *J* = 8.0 Hz, 1H), 7.26–7.10 (m, 5H), 7.07–7.02 (m, 1H), 6.06 (s, 1H), 4.29 (d, *J* = 8.5 Hz, 1H), 3.87 (s, 3H), 1.65–1.63 (m, 1H), 1.19 (s, 9H), 0.59 (dd, *J* = 20.8, 6.6 Hz, 6H). ^13^C NMR (75 MHz, Chloroform-*d*) δ 167.4, 160.5, 150.4, 145.1, 137.5, 136.8, 131.3, 130.5, 129.9, 125.2, 123.9, 121.5, 121.1, 120.9, 114.7, 111.5, 104.9, 82.5, 71.5, 55.8, 30.3, 27.9, 20.1, 19.0.HRMS Calcd. For C_30_H_33_N_3_NaO_7_S [M+Na]^+^: 602.19314; found: 602.1919.

*methyl N-(1-(2-methoxyphenyl)-1H-indol-2-yl)-N-((4-nitrophenyl)sulfonyl)-D-valinate***3n**. According to the general procedure, purification by flash chromatography, **3n** (30.1 mg, 28%, *dr* = 5:1) was obtained as a yellow solid from **1a** (0.2 mmol, 44.6 mg), **2n** (0.6 mmol, 189.8 mg), and NaClO (0.6 mmol, 774 μL, 5% in water). ^1^H NMR (300 MHz, Chloroform-*d*) δ 8.439 (d, *J =* 9.0 Hz, 2H × 0.83), 8.33 (d, *J =* 9.0 Hz, 2H × 0.17), 8.09 (d, *J* = 9.0 Hz, 2H × 0.83), 8.03 (d, *J =* 9.0 Hz, 2H × 0.17), 7.82 (d, *J* = 7.8 Hz, 1H), 7.55–7.47 (m, 2H), 7.21–7.02 (m, 4H), 6.83 (d, *J* = 8.4 Hz, 1H), 6.02 (s, 1H × 0.16), 5.96 (s, 1H × 0.83), 4.31 (d, *J* = 9.9 Hz, 1H), 3.70 (1H × 0.16), 3.67 (s, 9H × 0.83), 3.46 (s, 3H × 0.16), 3.36 (s, 3H × 0.83), 1.86–1.78 (m, 1H), 0.88–0.45 (m, 6H). ^13^C NMR (75 MHz, CDCl_3_) δ 168.7, 156.6, 150.5, 144.3, 132.5, 130.6, 125.1, 124.7, 121.3, 121.0, 120.2, 111.7, 111.4, 72.1, 55.6, 51.7, 28.6, 19.6. HRMS Calcd. For C_31_H_35_N_3_NaO_7_S [M+Na]^+^: 616.2088; found: 616.2074.

*ethyl N-(1-(2-methoxyphenyl)-1H-indol-2-yl)-N-((4-nitrophenyl)sulfonyl)-D-valinate***3o**. According to the general procedure, purification by flash chromatography, **3o** (44.1 mg, 40%, *dr* = 6:1) was obtained as a yellow solid from **1a** (0.2 mmol, 44.6 mg), **2o** (0.6 mmol, 198.2 mg), and NaClO (0.6 mmol, 774 μL, 5% in water). ^1^H NMR (300 MHz, Chloroform-*d*) δ 8.40–8.37 (m, 2H × 0.86), 8.40–8.37 (m, 2H × 0.14), 8.13–7.86 (m, 3H), 7.53–7.47 (m, 2H), 7.21–7.03 (m, 4H), 6.84 (d, *J* = 9.6 Hz, 1H), 6.03 (s, 1H × 0.14), 5.94 (s, 1H × 0.86), 4.30 (d, *J* = 10.0 Hz, 1H), 3.92–3.79 (m, 1H), 3.71 (s, 3H × 0.14), 3.67 (s, 3H × 0.86), 3.29 (t, *J* = 7.1 Hz, 1H), 1.87–1.71 (m, 1H), 1.16–0.89 (m, 3H), 0.80–0.43 (m, 6H). Crude ^13^C NMR (75 MHz, Chloroform-*d*) δ 168.7, 156.9, 150.9, 145.0, 137.4, 132.9, 131.6, 131.1, 128.6, 125.4, 125.1, 124.9, 124.1, 124.0, 121.7, 121.4, 120.9, 112.1, 111.9, 103.8, 61.4, 55.7, 42.7, 30.2, 29.1, 20.1, 19.9, 14.7, 14.2. HRMS Calcd. For C_28_H_29_N_3_NaO_7_S [M+Na]^+^: 574.1618; found: 574.1602.

*tert-butyl N-(1-(2-methoxyphenyl)-1H-indol-2-yl)-N-((4-nitrophenyl)sulfonyl)-D-isoleucinate* **3p**. According to the general procedure, purification by flash chromatography, **3p** (49.9 mg, 42%, *dr* = 25:1) was obtained as a yellow solid from **1a** (0.2 mmol, 44.6 mg), **2p** (0.6 mmol, 223.5 mg), and NaClO (0.6 mmol, 774 μL, 5% in water). ^1^H NMR (300 MHz, Chloroform-*d*) δ 8.37 (d, *J* = 8.7 Hz, 2H), 8.07 (d, *J* = 8.7 Hz, 2H), 8.00 (d, *J* = 7.9 Hz, 1H), 7.56–7.47 (m, 2H), 7.21–7.07 (m, 4H), 6.84 (d, *J* = 8.3 Hz, 1H), 6.27 (s, 1H), 4.40 (d, *J* = 6.6 Hz, 1H), 3.67 (s, 3H), 1.50–1.42 (m, 1H), 1.23 (s, 9H), 1.09–0.88 (m, 2H), 0.57–0.48 (m, 6H).^13^C NMR (101 MHz, Chloroform-*d*) δ 167.9, 156.0, 150.3, 145.0, 136.9, 132.2, 131.1, 130.6, 130.3, 125.0, 124.8, 123.7, 123.4, 121.3, 121.0, 120.4, 111.9, 111.5, 105.2, 82.2, 69.4, 55.2, 35.7, 28.0, 27.2, 15.7, 11.5.HRMS Calcd. For C_31_H_35_N_3_NaO_7_S [M+Na]^+^: 616.2088; found: 616.2076.

*methyl (R)-2-((N-(1-(2-methoxyphenyl)-1H-indol-2-yl)-4-nitrophenyl)sulfonamido)pentanoate***3q**. According to the general procedure, purification by flash chromatography, **3q** (30.1 mg, 28%, *dr* = 8:1) was obtained as a yellow solid from **1a** (0.2 mmol, 44.6 mg), **2q** (0.6 mmol, 189.8 mg), and NaClO (0.6 mmol, 774 μL, 5% in water). ^1^H NMR (300 MHz, Chloroform-*d*) δ 8.41 (d, *J* = 8.8 Hz, 2H), 8.09 (d, *J* = 8.8 Hz, 2H), 7.78–7,75 (m, 1H), 7.7–7.51 (m, 2H), 7.27–7.16 (m, 3H), 7.09 (d, *J* = 7.5 Hz, 1H), 6.89 (d, *J* = 7.5 Hz, 1H), 6.38 (s, 1H × 0.89), 6.27 (s, 1H × 0.11), 4.61 (t, *J* = 7.4 Hz, 1H), 3.71 (s, 3H × 0.89), 3.70 (s, 1H × 0.11), 3.52 (s, 3H × 0.11), 3.43 (s, 3H × 0.89), 1.88–0.72 (m, 6H). ^13^C NMR (75 MHz, Chloroform-*d*) δ 170.8, 170.0, 156.0, 150.3, 144.6, 136.9, 132.0, 131.3, 130.5, 130.3, 125.2, 124.6, 123.8, 123.6, 123.6, 121.2, 121.0, 120.7, 120.6, 111.7, 111.5, 111.5, 103.8, 64.4, 55.4, 55.2, 52.4, 52.0, 32.0, 20.1, 19.3, 14.3, 13.9. HRMS Calcd. For C_27_H_27_N_3_NaO_7_S [M+Na]^+^: 560.1462; found: 560.1452.

*methyl N-(1-(2-methoxyphenyl)-1H-indol-2-yl)-N-((4-nitrophenyl)sulfonyl)-D-alaninate***3r**. According to the general procedure, purification by flash chromatography, **3r** (28.5 mg, 28%, *dr* = 6:1) was obtained as a yellow solid from **1a** (0.2 mmol, 44.6 mg), **2r** (0.6 mmol, 173.0 mg), and NaClO (0.6 mmol, 774 μL, 5% in water). ^1^H NMR (300 MHz, Chloroform-*d*) δ 8.34–8.24 (m, 2H), 7.99 (d, *J* = 9.0 Hz, 2H × 0.86), 7.91 (d, *J =* 9.0 Hz, 2H × 0.13), 7.66–7.46 (m, 3H), 7.20–7.02 (m, 4H), 6.86 (d, *J* = 8.1 Hz, 1H), 6.51 (s, 1H × 0.86), 6.33 (1H × 0.14), 4.73 (q, *J* = 7.3 Hz, 1H), 3.63 (s, 3H), 3.58 (s, 3H × 0.14), 3.48 (s, 3H × 0.86), 1.19 (d, *J* = 7.3 Hz, 3H). ^13^C NMR (75 MHz, CDCl_3_) δ 170.7, 156.0, 150.3, 144.8, 137.0, 131.8, 130.4, 125.3, 124.6, 123.7, 121.2, 120.6, 111.8, 111.4, 104.4, 55.3, 52.4, 15.7. HRMS Calcd. For C_25_H_23_N_3_NaO_7_S [M+Na]^+^: 532.1149; found: 532.1137.

*tert-butyl (R)-2-cyclohexyl-2-((N-(1-(2-methoxyphenyl)-1H-indol-2-yl)-4-nitrophenyl)sulfonamido)acetate***3s** and *tert-butyl (R)-2-((N-(3-chloro-1-(2-methoxyphenyl)-1H-indol-2-yl)-4-nitrophenyl)sulfonamido)-2-cyclohexylacetate* **4s**. According to the general procedure, purification by flash chromatography, **3s** (48.3 mg, 39%, *dr* = 5:1) and **4s** (34.0 mg, 26%, >20:1) was obtained as a yellow solid from **1a** (0.2 mmol, 44.6 mg), **2s** (0.6 mmol, 239.1 mg), and NaClO (0.6 mmol, 774 μL, 5% in water). ^1^H NMR (300 MHz, Chloroform-*d*) δ 8.32–8.25 (m, 2H × 0.83), 8.25–8.21 (m, 2H × 0.17), 8.08–8.05 (m, 2H × 0.83), 8.08–8.05 (m, 2H × 0.17),8.05–7.93 (m, 1H × 0.55, from **4s**), 7.84–7.82 (m, 1H), 7.62–7.59 (m, 1H), 7.43–7.17 (m, 5H), 7.14–6.93 (m, 8H), 6.76–6.73 (m, 1.5 H), 6.46–6.43 (m, 1 H × 0.55 from 4s), 5.96 (s, 1H × 0.17), 5.71 (s, 1H × 0.83), 4.20 (m, 1H), 3.71 (s, 3H × 0.55 from **4s**), 3.65 (s, 3H × 0.83), 3.65 (s, 3H × 0.17), 1.63–13.2 (m, 9H), 1.10(s, 9H × 0.55, from **4s**), 1.09 (s, 9H × 0.17), 1.07 (s, 9H × 0.83), 0.90–0.42 (m, 8H). ^13^C NMR (75 MHz, CDCl_3_) δ 168.4, 167.0, 156.0, 155.5, 150.3, 145.0, 141.4, 136.7, 132.3, 131.8, 131.1, 131.0, 130.6, 130.3, 129.4, 129.1, 124.8, 124.8, 124.6, 124.2, 123.8, 123.5, 123.4, 121.4, 121.3, 121.2, 120.9, 120.8, 120.5, 120.4, 112.7, 111.7, 111.7, 110.6, 104.2, 82.2, 72.3, 56.0, 55.5, 55.2, 37.7, 30.2, 29.9, 27.9, 27.7, 26.3, 26.2, 26.1. HRMS Calcd. For C_33_H_37_N_3_NaO_7_S [M+Na]^+^: 642.2244; found: 642.2231.

*(R)-N-(1-((tert-butyldimethylsilyl)oxy)propan-2-yl)-N-(1-(2-methoxyphenyl)-1H-indol-2-yl)-4-nitrobenzenesulfonamide* **3t** and *(R)-N-(1-((tert-butyldimethylsilyl)oxy)propan-2-yl)-N-(3-chloro-1-(2-methoxyphenyl)-1H-indol-2-yl)-4-nitrobenzenesulfonamide* **4t**. According to the general procedure, purification by flash chromatography, **3t** (44.1 mg, 37%, *dr* > 20:1) and **4t** (23.3 mg, 19%, *dr* > 20:1) was obtained as a yellow solid from **1a** (0.2 mmol, 44.6 mg), **2v** (0.6 mmol, 224.7 mg), and NaClO (0.6 mmol, 774 μL, 5% in water). Crude ^13^H NMR of **3t** and **4t** (300 MHz, Chloroform-*d*) δ 8.44–8.34 (m, 2H, from **3t**), 8.44–8.34 (m, 1H, from **4t**), 8.17–8.09 (m, 3H, from **3t** and **4t**), 7.91–7.84 (m, 1H from **3t**, 0.5H from 4t), 7.62–7.47 (m, 2H from **3t**, 1H from **4t**), 7.20–7.05 (m, 4H from **3t,** 2H from **4t**), 6.8 (m, 1H from **3t**, 0.5H from **4t**), 6.03 (s, 1H, from **3t**), 4.08–3.87 (m, 1H from **3t**, 0.5H from **4t**), 3.70 (s, 3H from **3t**), 3.67 (s, 1.5H from **4t**), 3.43–2.5 (m, 2H from **3t**, 1H from **4t**), 0.96–0.86 (m, 3H from **3t**, 1.5H from **4t**), 0.78 (s, 9H from **3t**, 4,5H from **4t**), −0.11 (s, 6H from **3t**), −0.14 (s, 3H from **4t**). Crude ^13^C NMR of **3t** and **4t** (75 MHz, Chloroform-*d*) δ 156.3, 155.8, 150.4, 146.7, 145.5, 136.5, 135.6, 132.2, 131.9, 130.9, 130.3, 130.2, 130.0, 129.6, 127.7, 125.1, 124.8, 124.5, 124.5, 124.3, 124.1, 123.5, 123.4, 121.5, 121.2, 121.1, 120.9, 120.6, 118.8, 112.0, 111.8, 111.7, 111.4, 103.2, 77.4, 65.9, 65.4, 61.4, 58.9, 55.5, 55.4, 25.9, 25.8, 18.2, 16.4, 15.7, −5.3, −5.4, −5.4. HRMS of **3t** Calcd. For C_30_H_37_N_3_NaO_6_SSi [M+Na]^+^: 618.2065; found: 618.2068. HRMS of **4t** Calcd. For C_30_H_36_ClN_3_NaO_6_SSi [M+Na]^+^: 652.1675; found: 652.1687.

## Figures and Tables

**Table 1:** Solvent screening for indole C–N axially chiral construction.

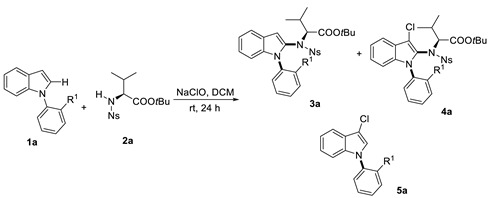			
Entry	Solvent	Yield/%^a^	*Dr*
1	DCM	65 (10 ^b^)	3.5/1 (15/1 ^c^)
2	DCE	55	2.4/1
3	PhCF_3_	38	2.5/1
4	MeOH	<5	-/-
5	PhMe	28	2.2/1
6	1,4-dioxane	18	2.5/1

^a^ reaction conditions: indoles (0.2 mmol), amino acid (0.6 mmol), 5% NaClO (0.6 mmol), DCM (4 mL), rt, 24 h; ^b^ yield of **4a**; ^c^ *dr* value of **4a**.

**Table 2:** Protecting group screening for indole C–N axially chiral construction.

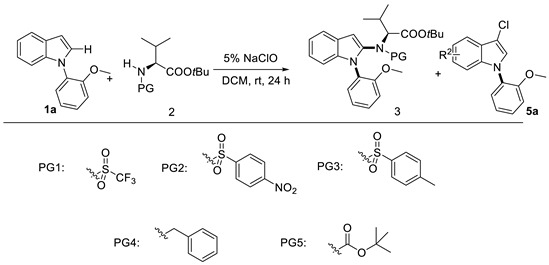			
Entry	Protecting Group	Yield/%^a^	Dr
1	PG1	<5	3.5/1
2	PG2	65	2.4/1
3	PG3	<5	2.5/1
4	PG4	<5 (60^b^)	-/-
5	PG5	<5 (71^b^)	-/-

^a^ reaction conditions: indoles (0.2 mmol), amino acid (0.6 mmol), 5% NaClO (0.6 mmol), DCM (4 mL), rt, 24 h. ^b^ yield of 5a.

## Data Availability

Not applicable.

## Supplementary Materials

^1^H and ^13^ C NMR of all compounds.can be downloaded at: https://www.mdpi.com/article/10.3390/molecules27249008/s1.

## Sample Availability

Samples of the compounds are available from the authors.
