# Supplementary material for: Atroposelective Amination of Indoles via Chiral Center Induced Chiral Axis Formation
Source: Molecules. 2022 Dec 17;27(24):9008. doi: 10.3390/molecules27249008 (PMC9783779; doi:10.3390/molecules27249008)
Supplement: Supplementary file 1 [file molecules-27-09008-s001.zip › molecules-2030224-supplementary.pdf]

Supporting Information

Atroposelective Amination of Indoles via Central-to-Axial Chirality Conversion Strategy

Yong Wang, Jingxue Yan, Yiqing Jiang, Zexuan Wei, Zhenlin, Tu, Chao Dong, Tao Lu, Yadong Chen*, Jie Feng*

Key Laboratory of Natural Medicines, Department of Organic Chemistry,
China Pharmaceutical University, Nanjing 210009, China
.

Supporting Information

1. NMR spectra for all compounds S2
2. X-ray date of compound **2a** S22
3. Stability experiments S23
4. DFT calculations S23

1. **NMR spectra for all compounds**

^{1}H NMR and ^{13}C NMR of **3a**

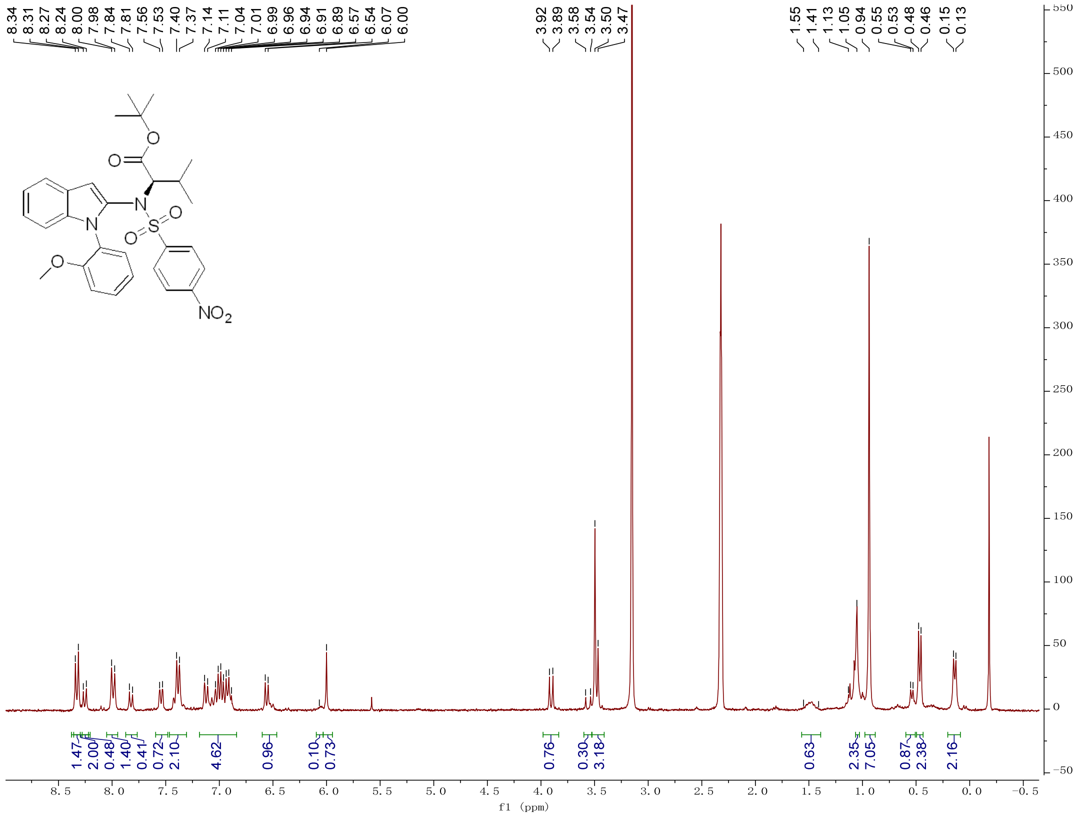

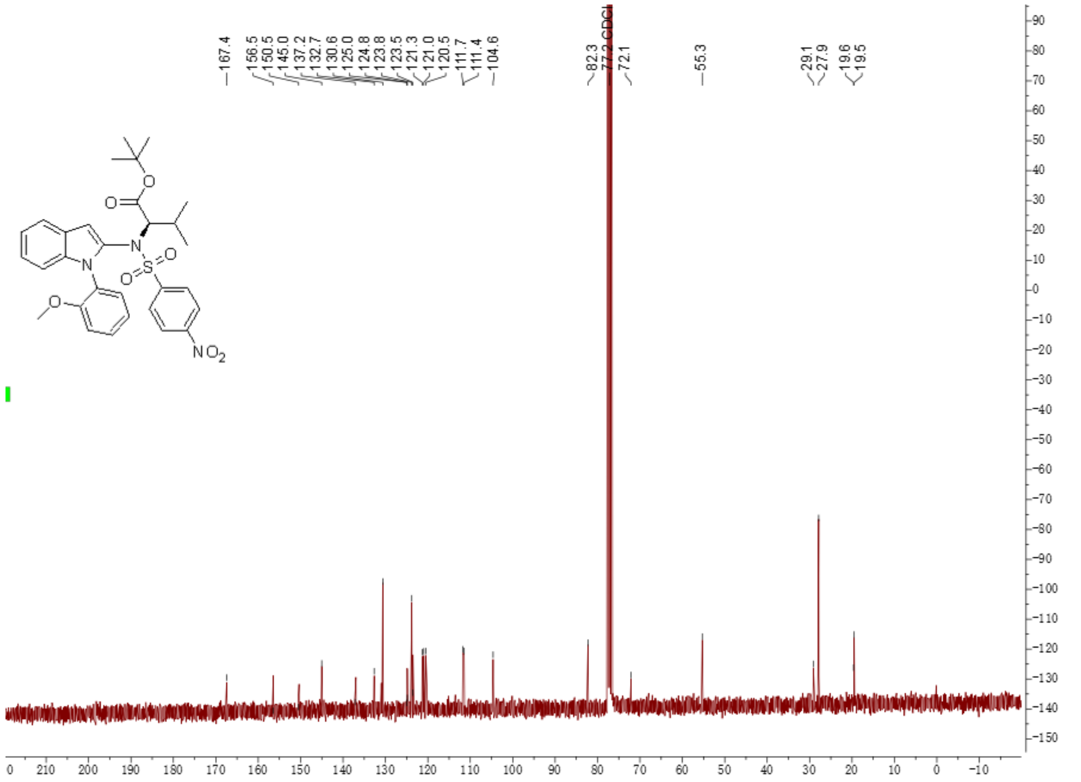

^{1}H NMR and ^{13}C NMR of **3b**

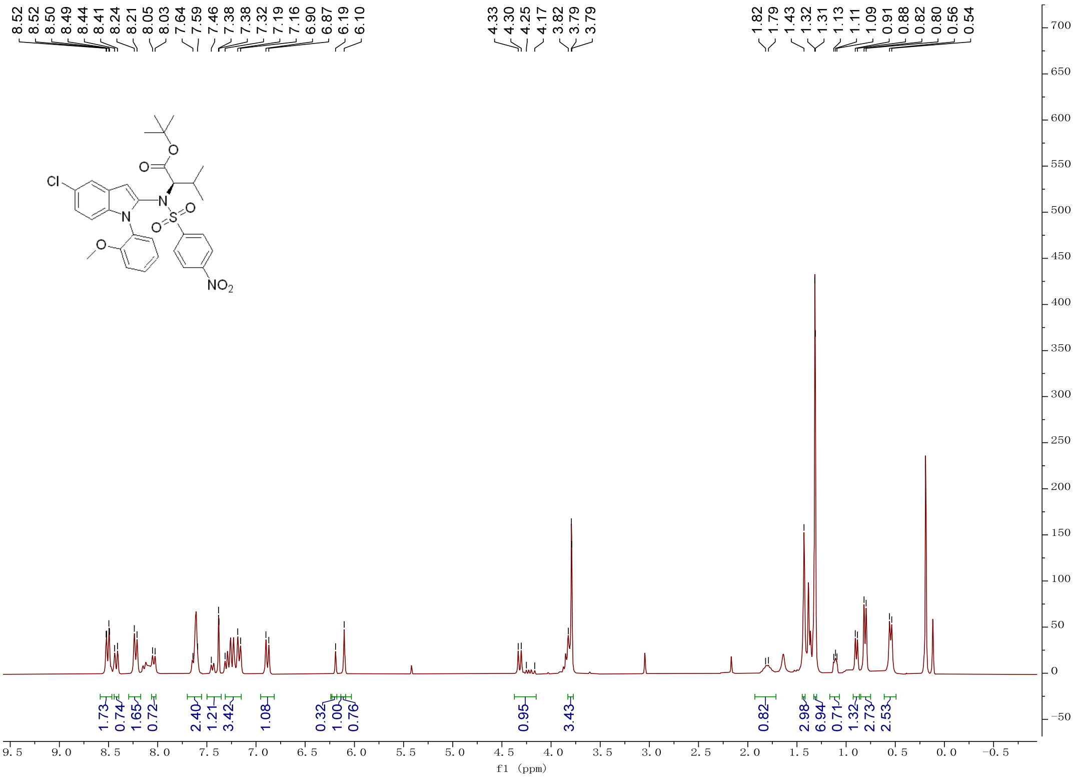

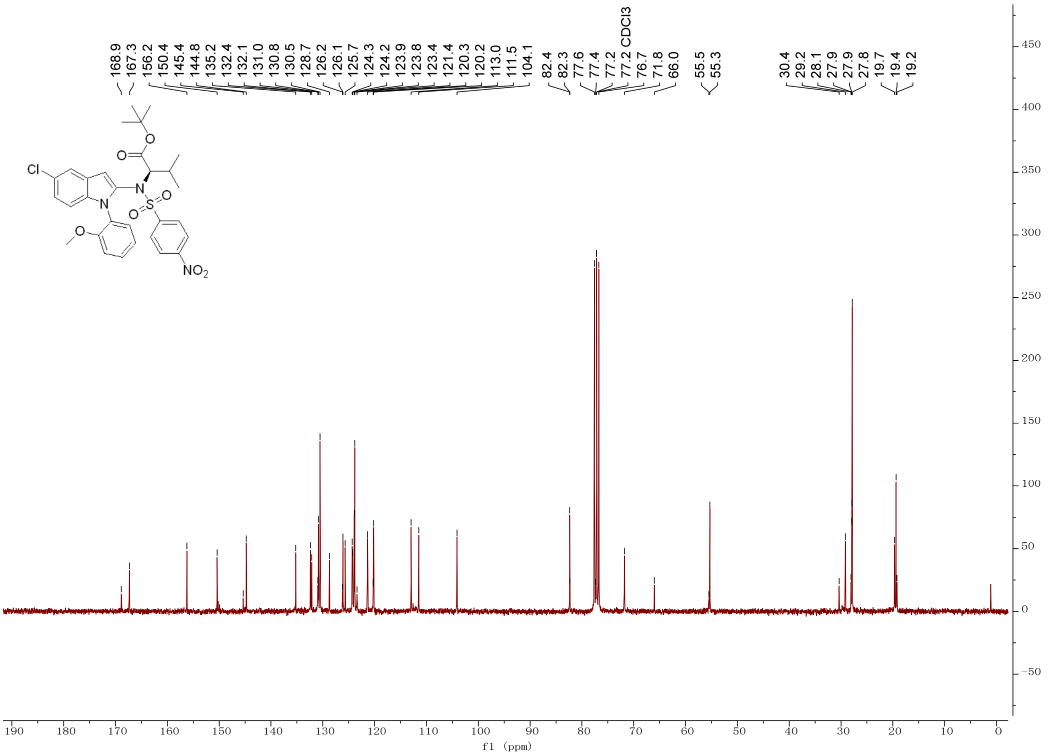

^{1}H NMR and ^{13}C NMR of **3c**

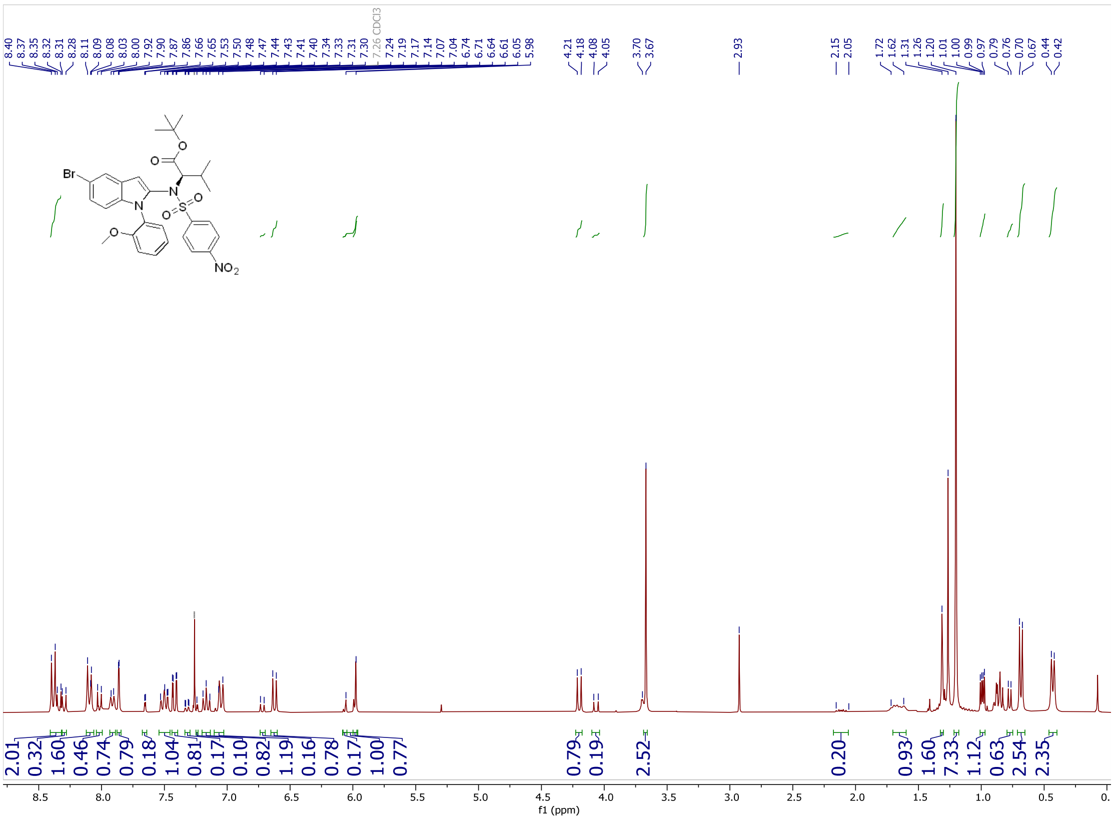

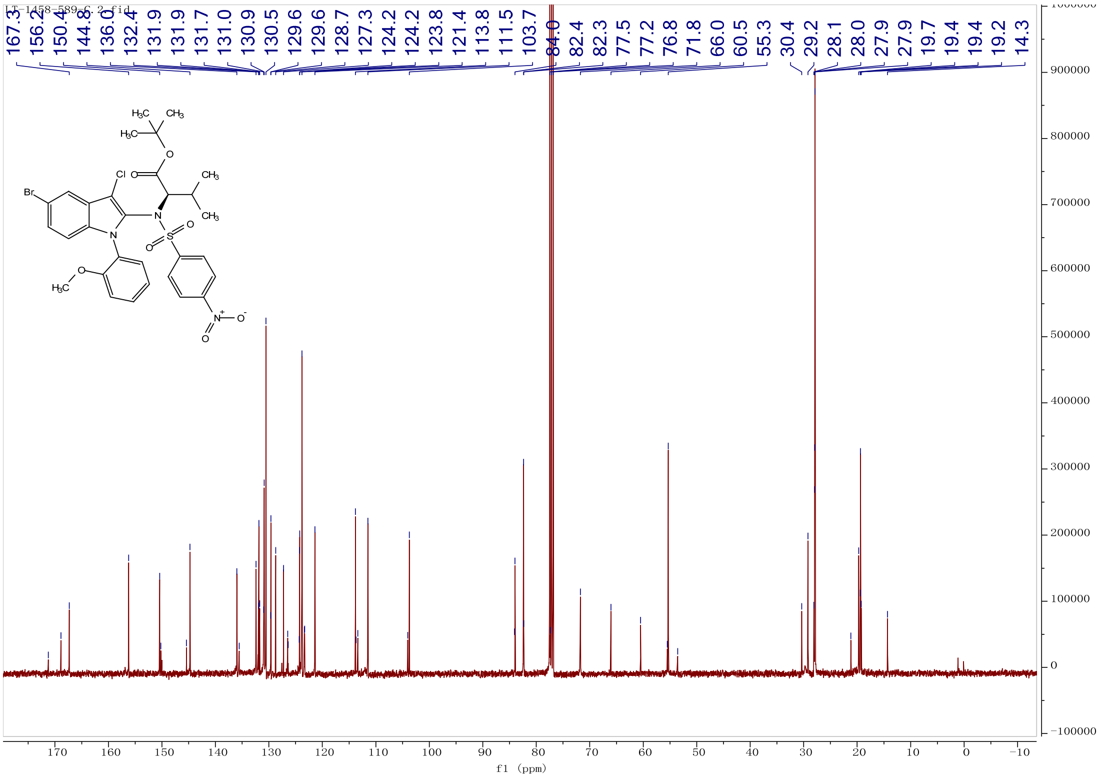

^{1}H NMR and ^{13}C NMR of **3d**

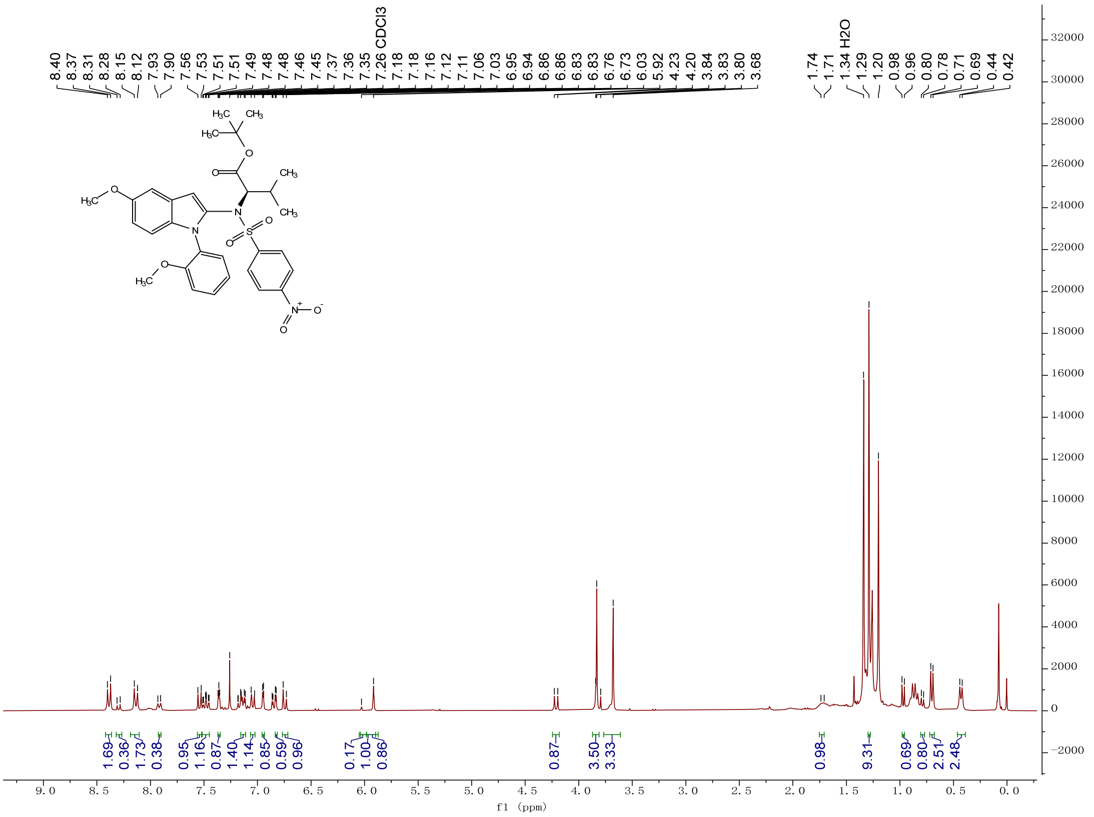

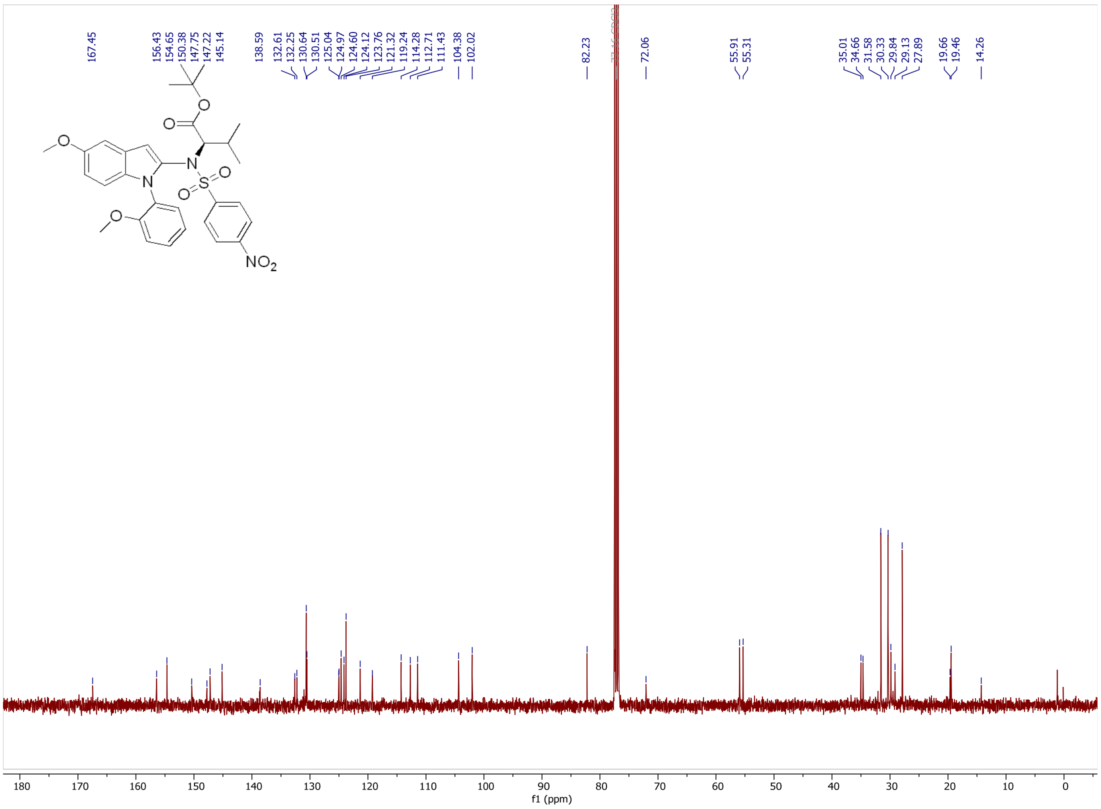

^{1}H NMR and ^{13}C NMR of **3e**

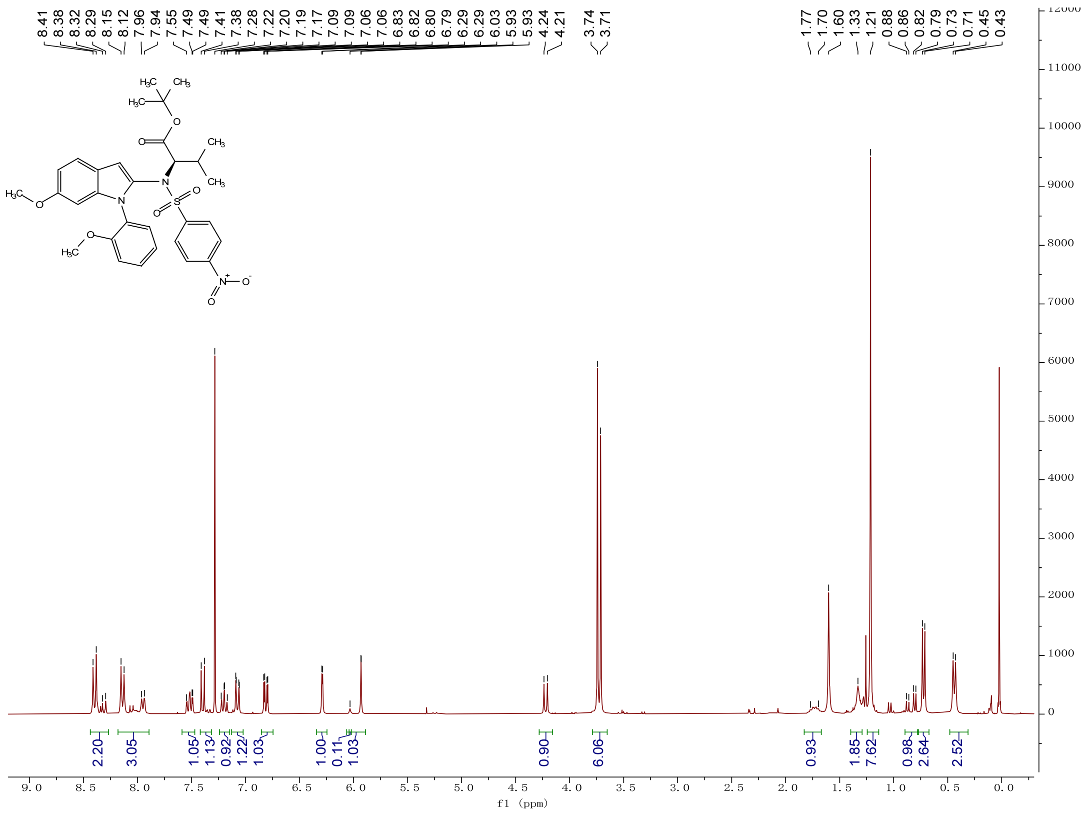

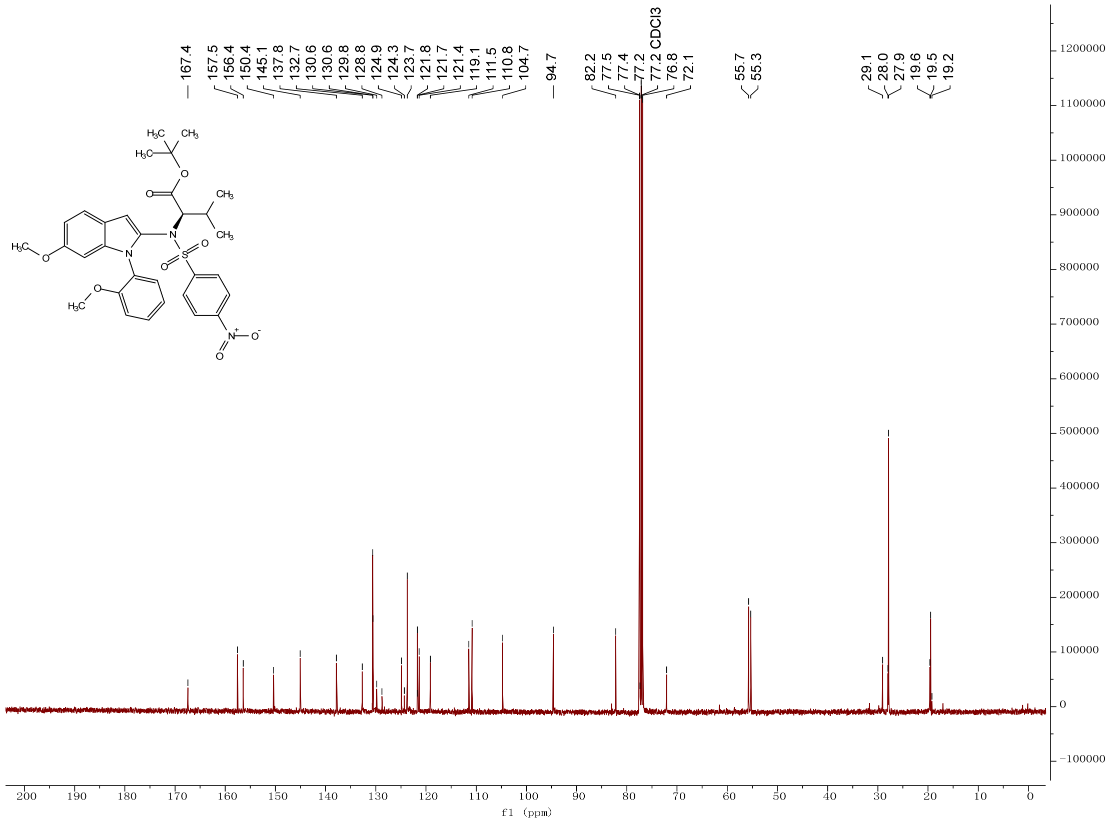

^{1}H NMR and ^{13}C NMR of **3f**

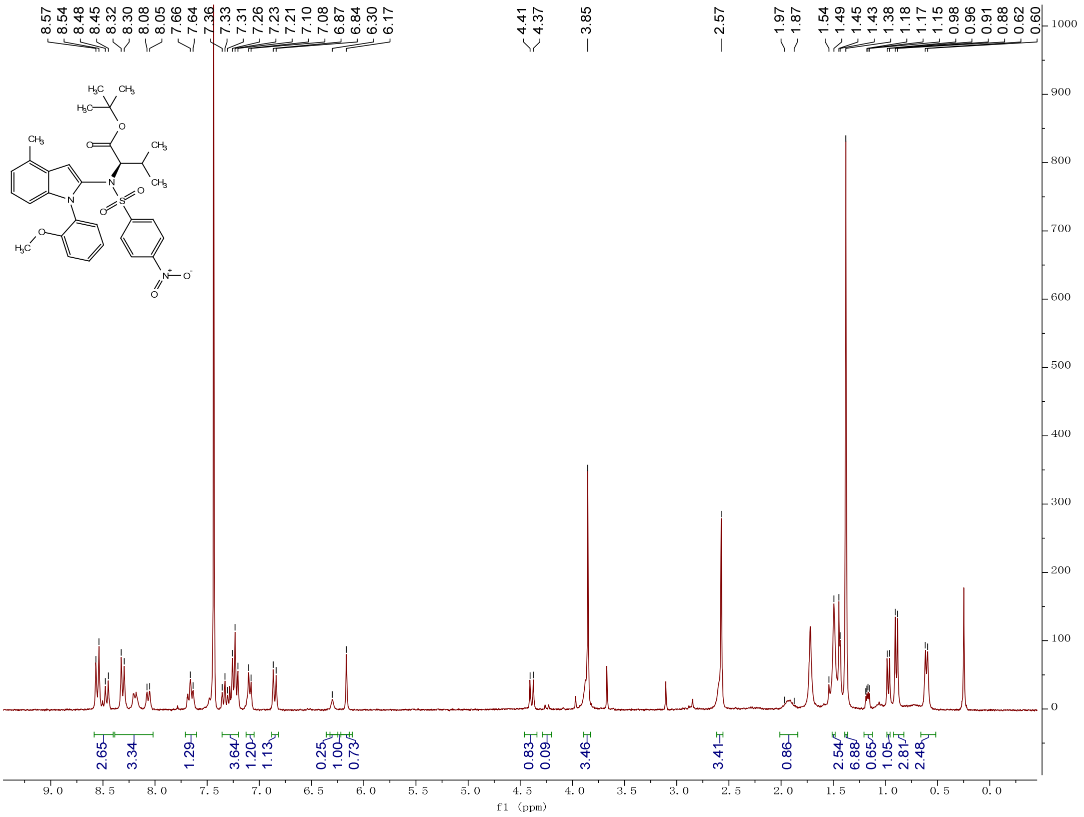

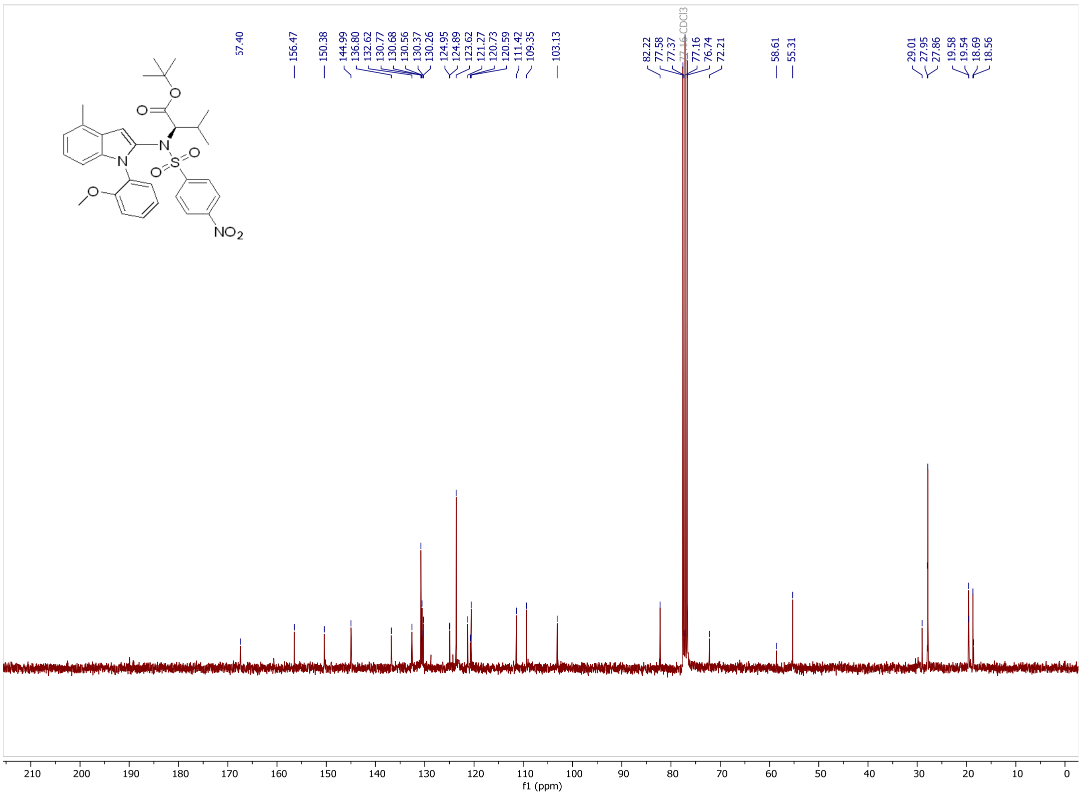

^{1}H NMR and ^{13}C NMR of **3g** and **4g**

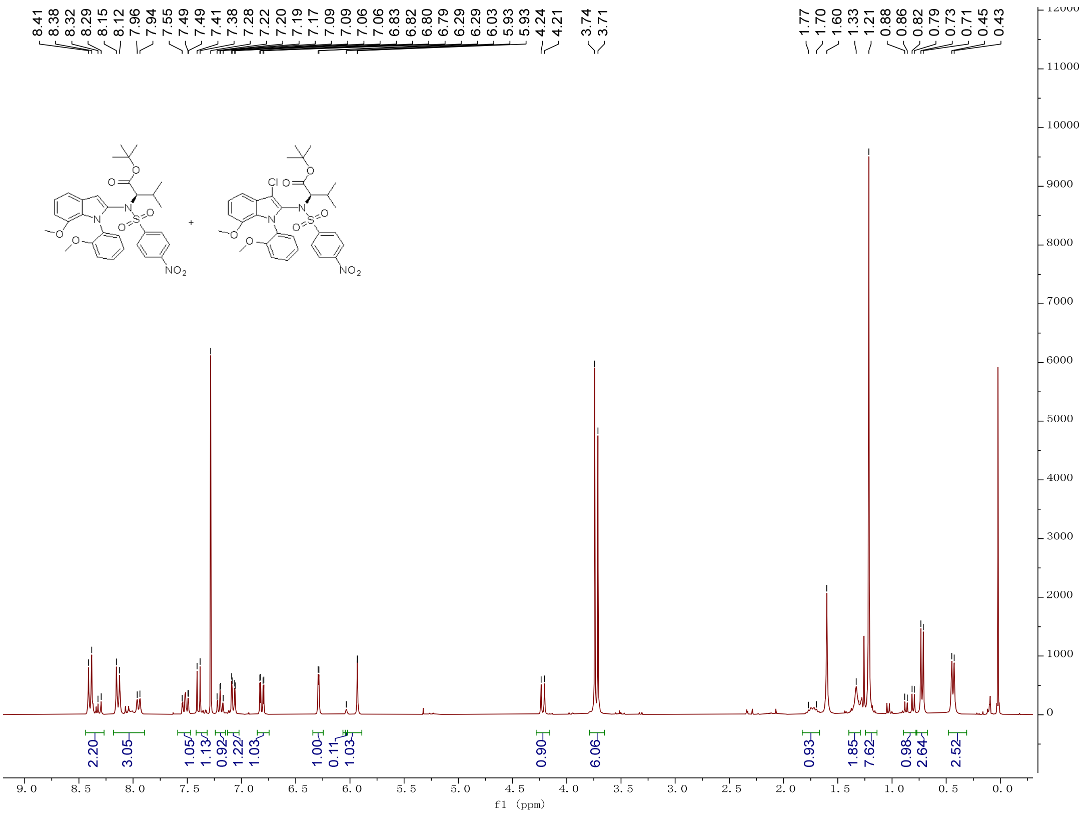

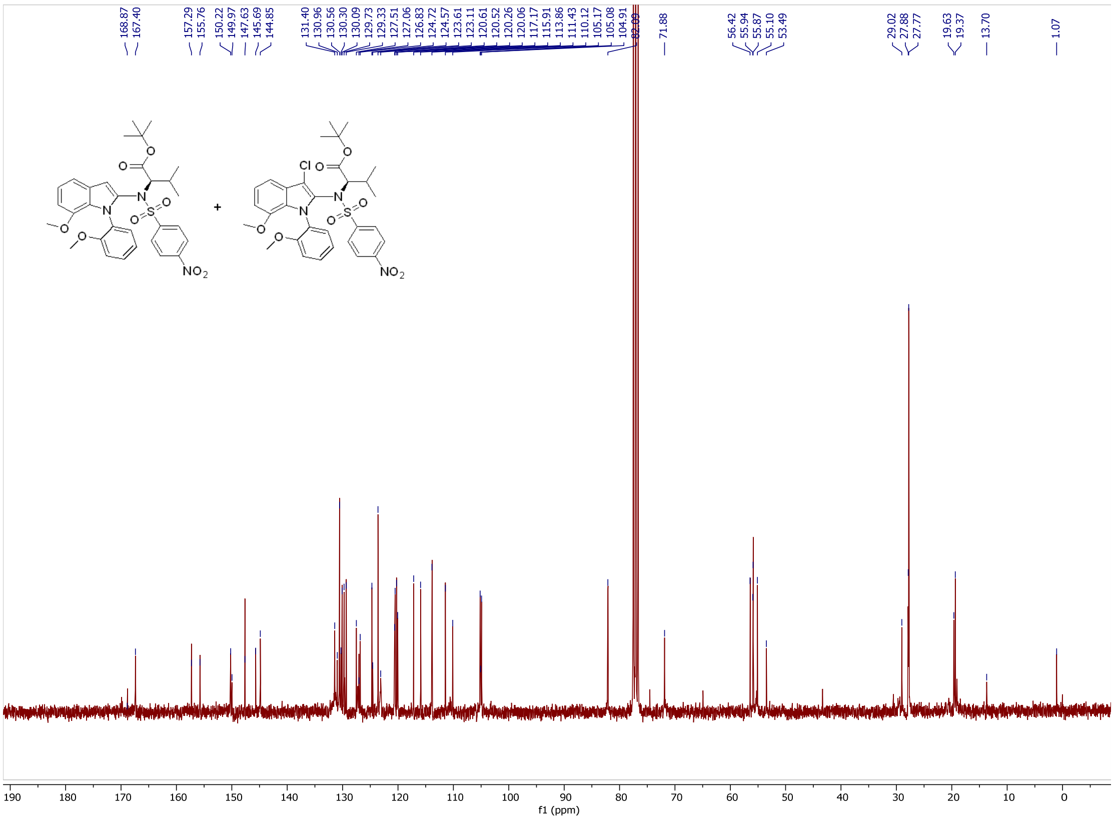

^{1}H NMR and ^{13}C NMR of **3h**

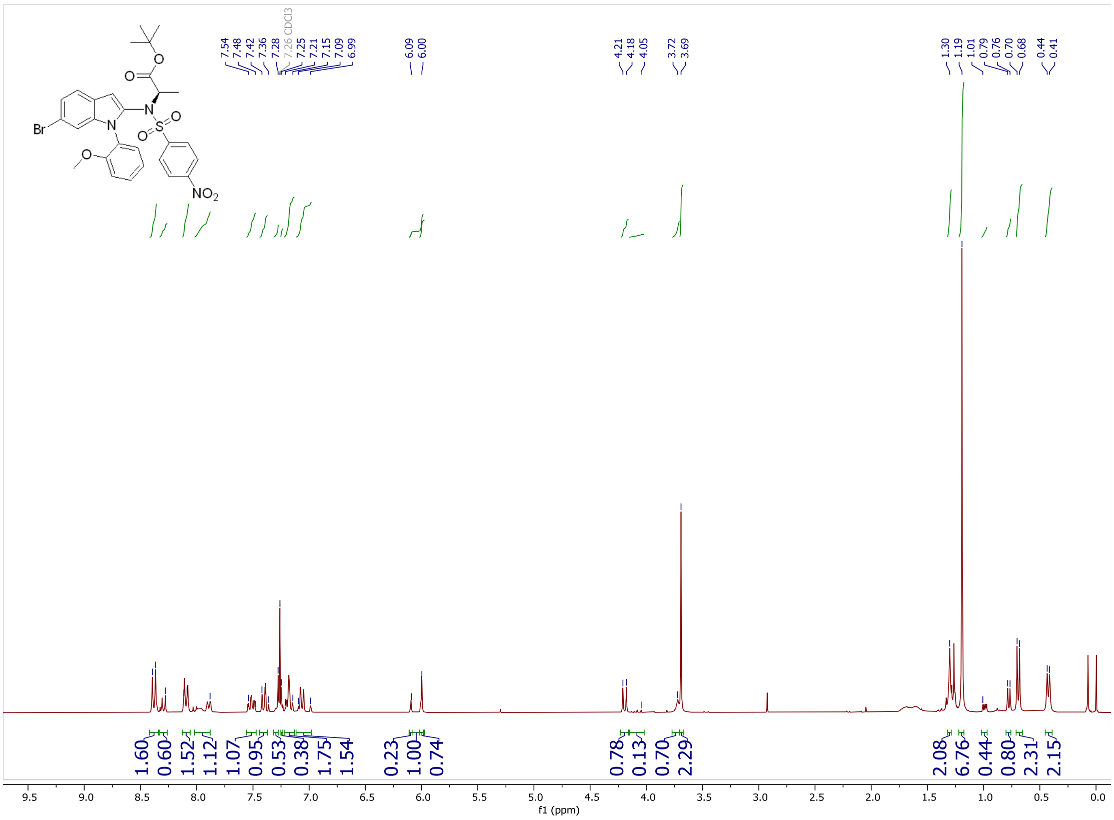

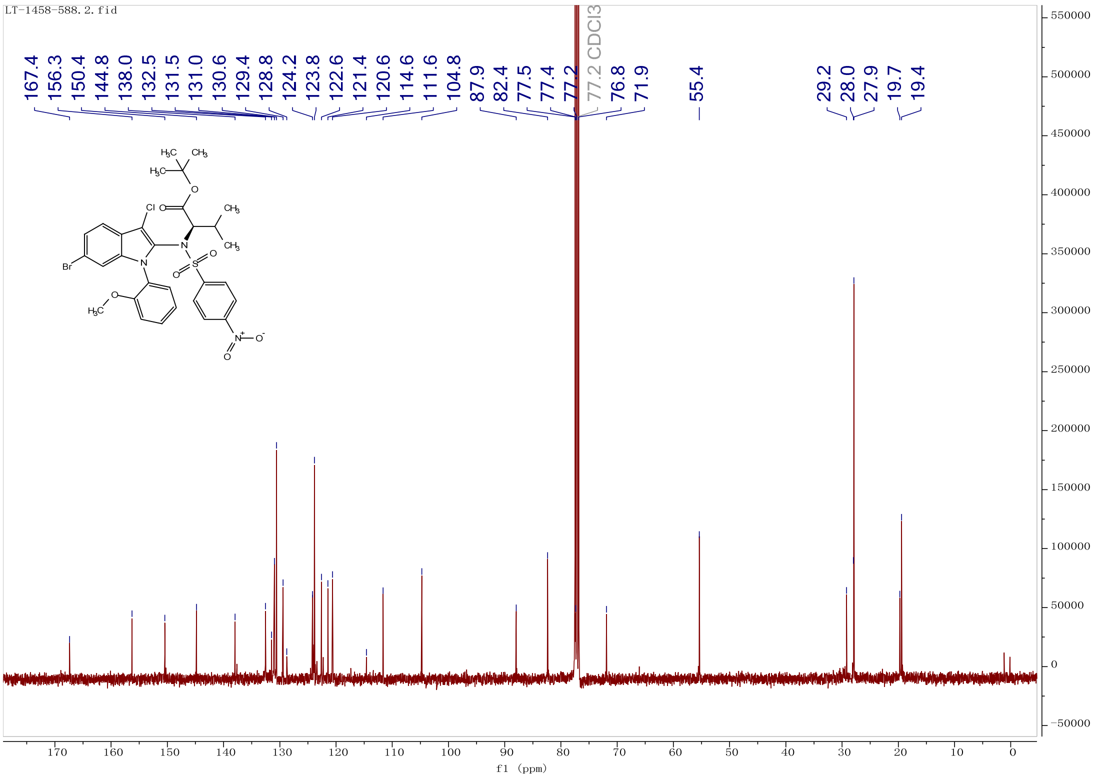

^{1}H NMR and ^{13}C NMR of **3i**

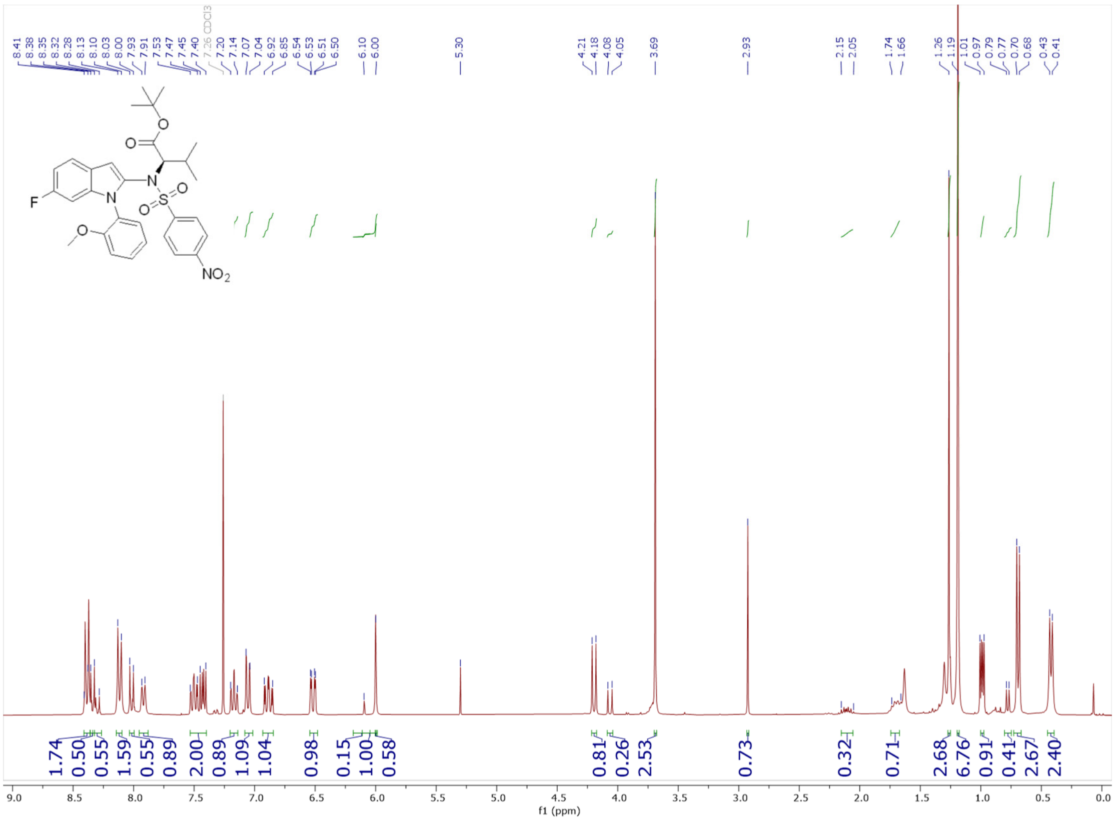

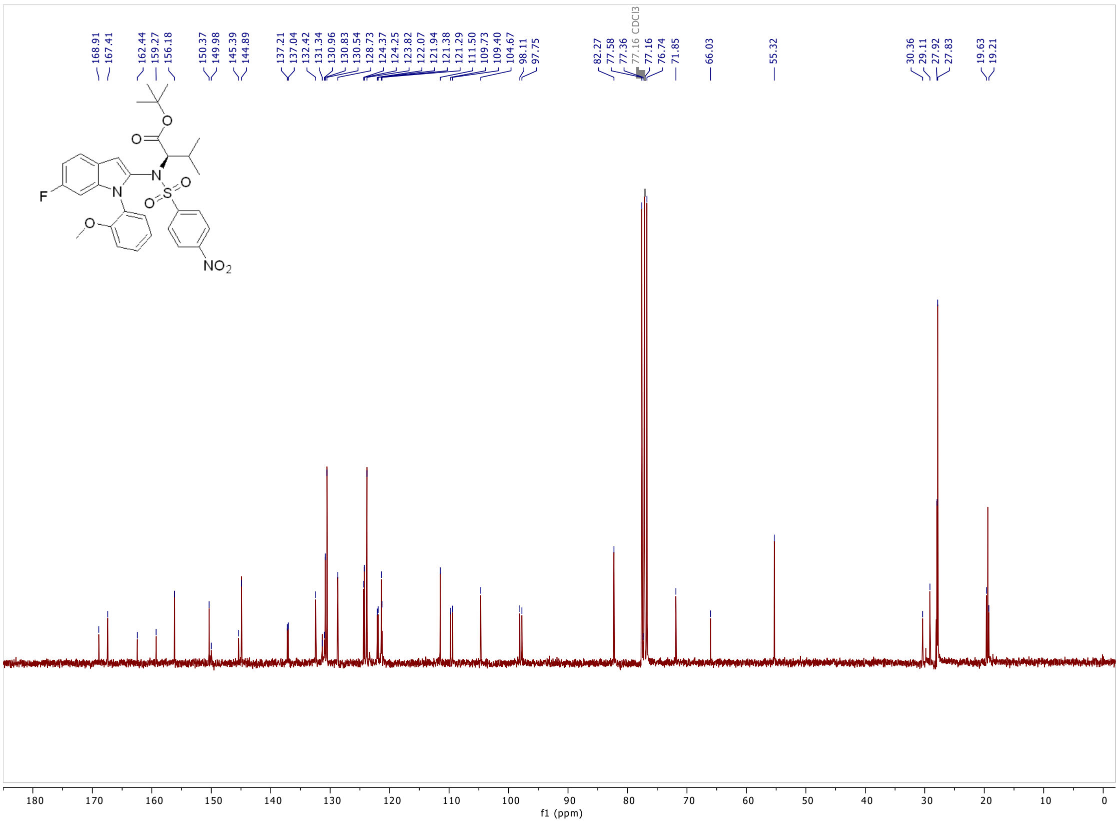

^{1}H NMR and ^{13}C NMR of **3j** and **4j**

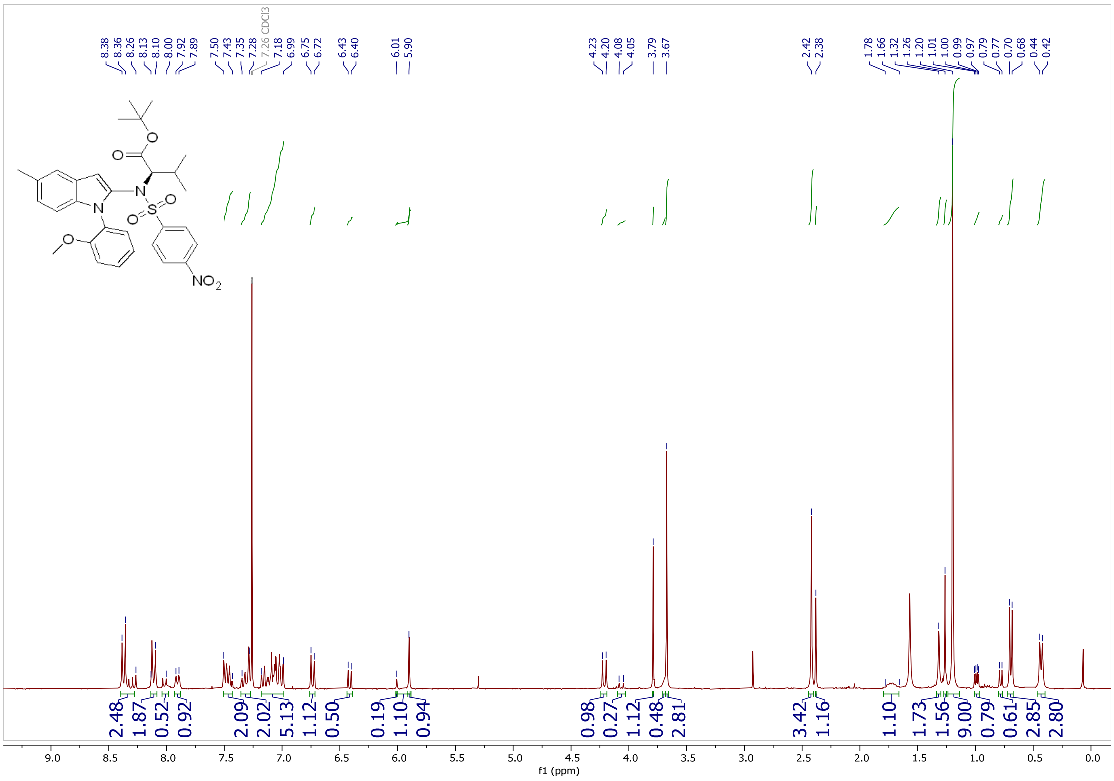

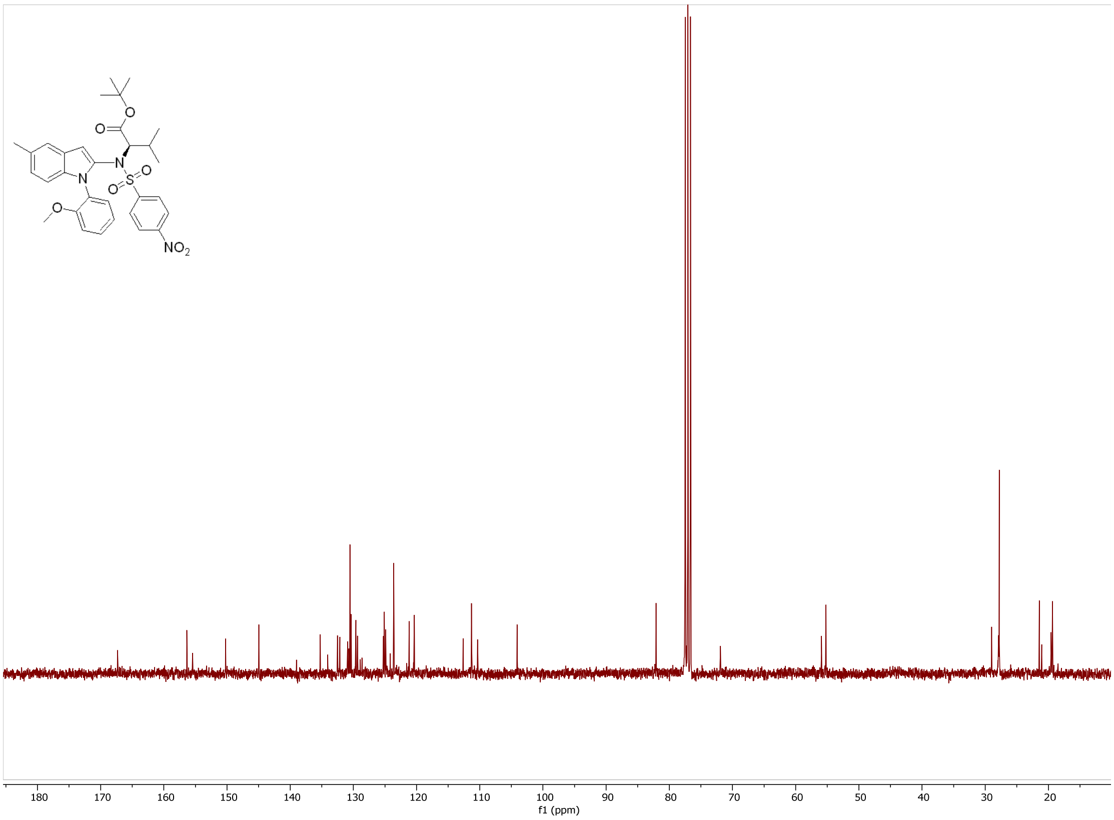

^{1}H NMR and ^{13}C NMR of **3k**

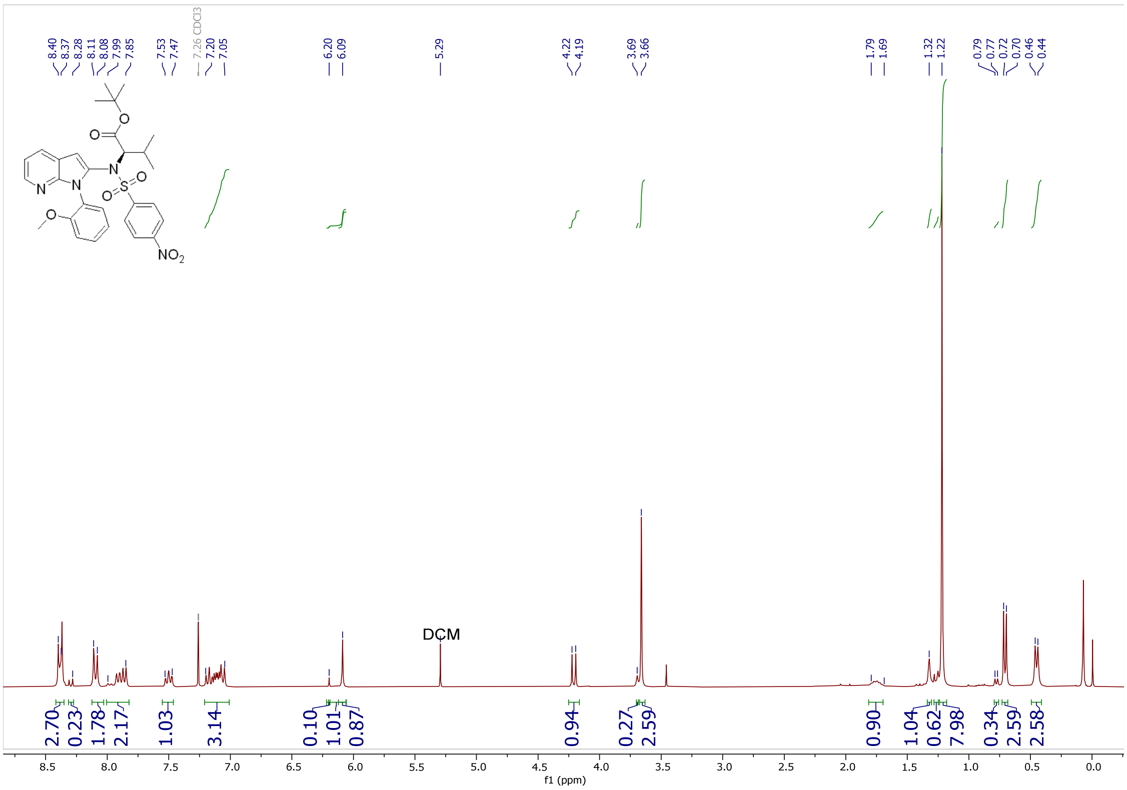

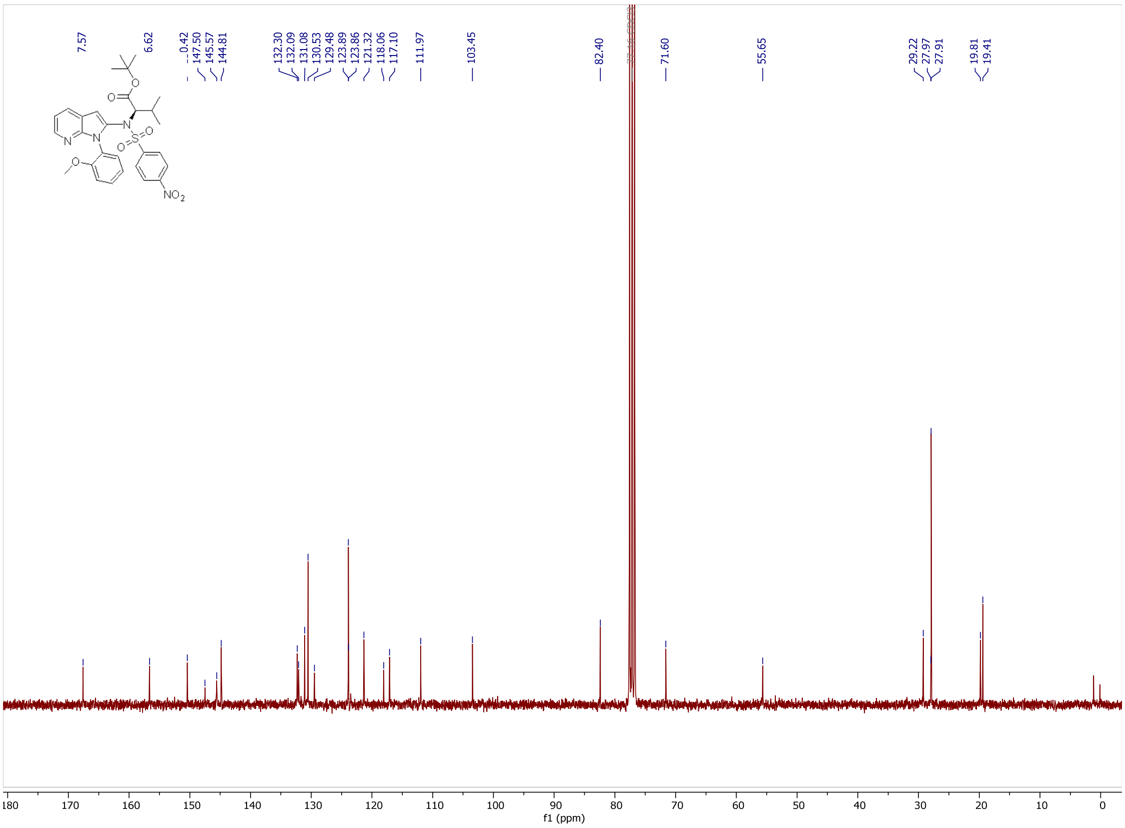

^{1}H NMR and ^{13}C NMR of **3l**

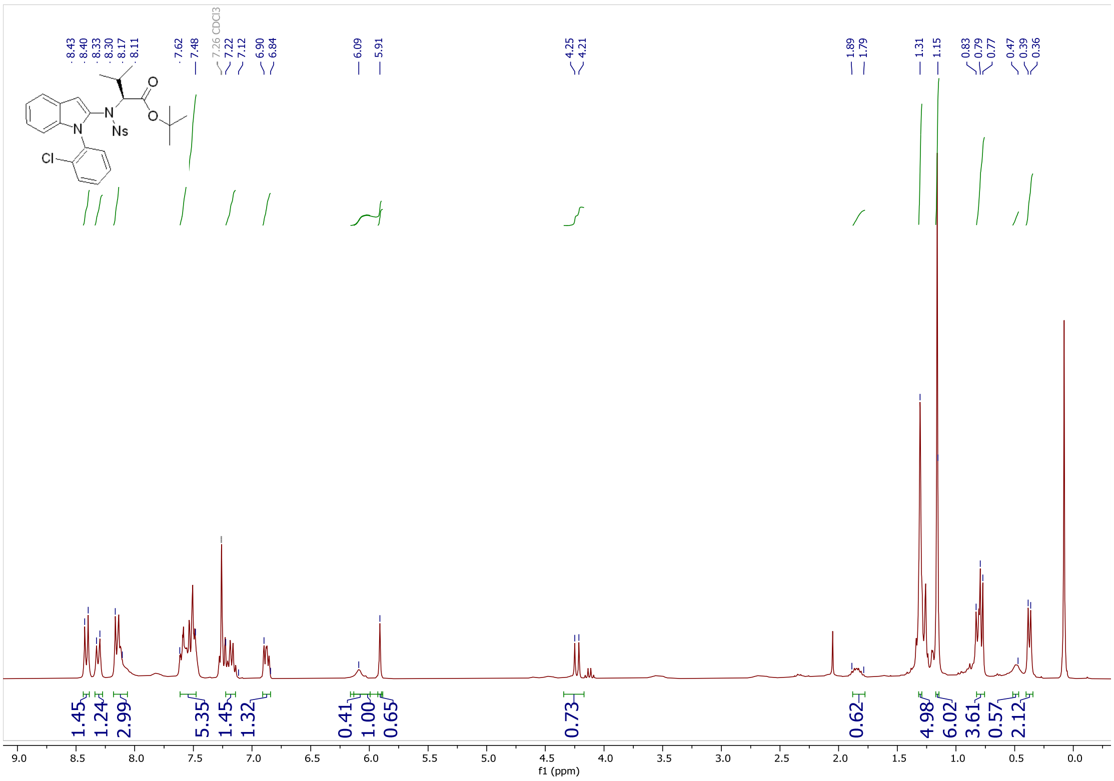

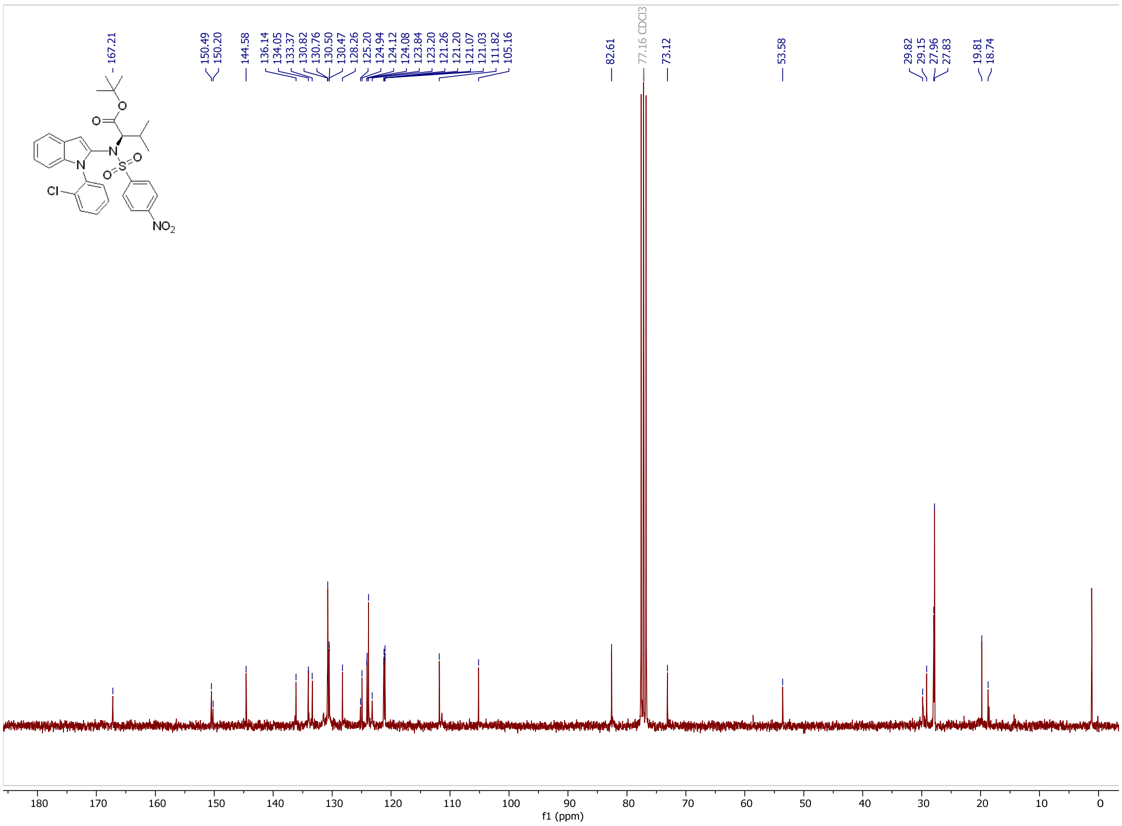

^{1}H NMR and ^{13}C NMR of **3m**

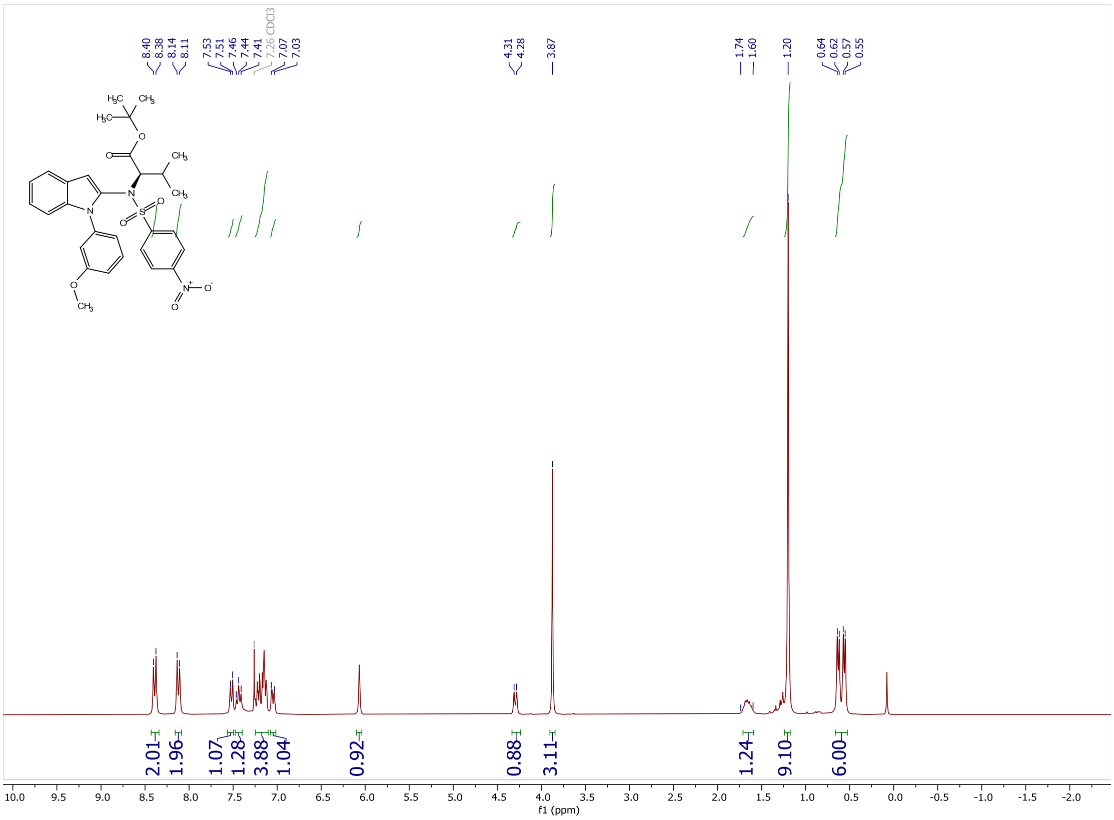

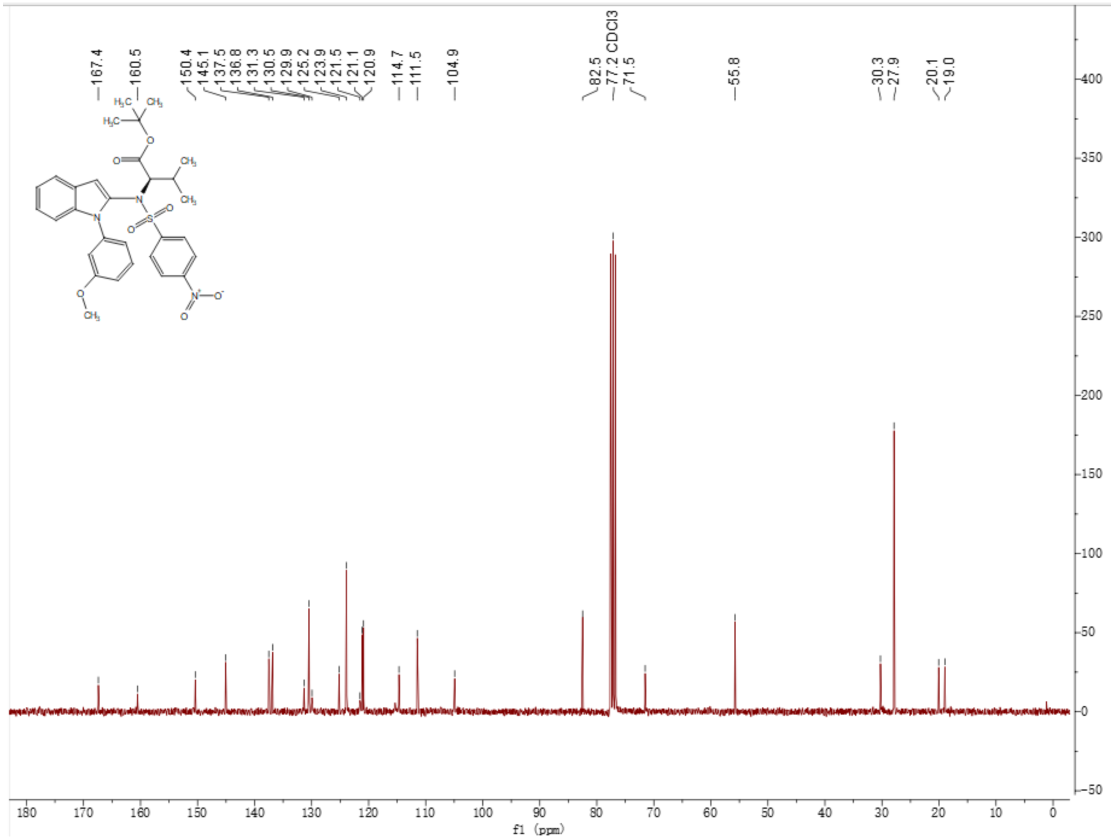

^{1}H NMR and ^{13}C NMR of **3n**

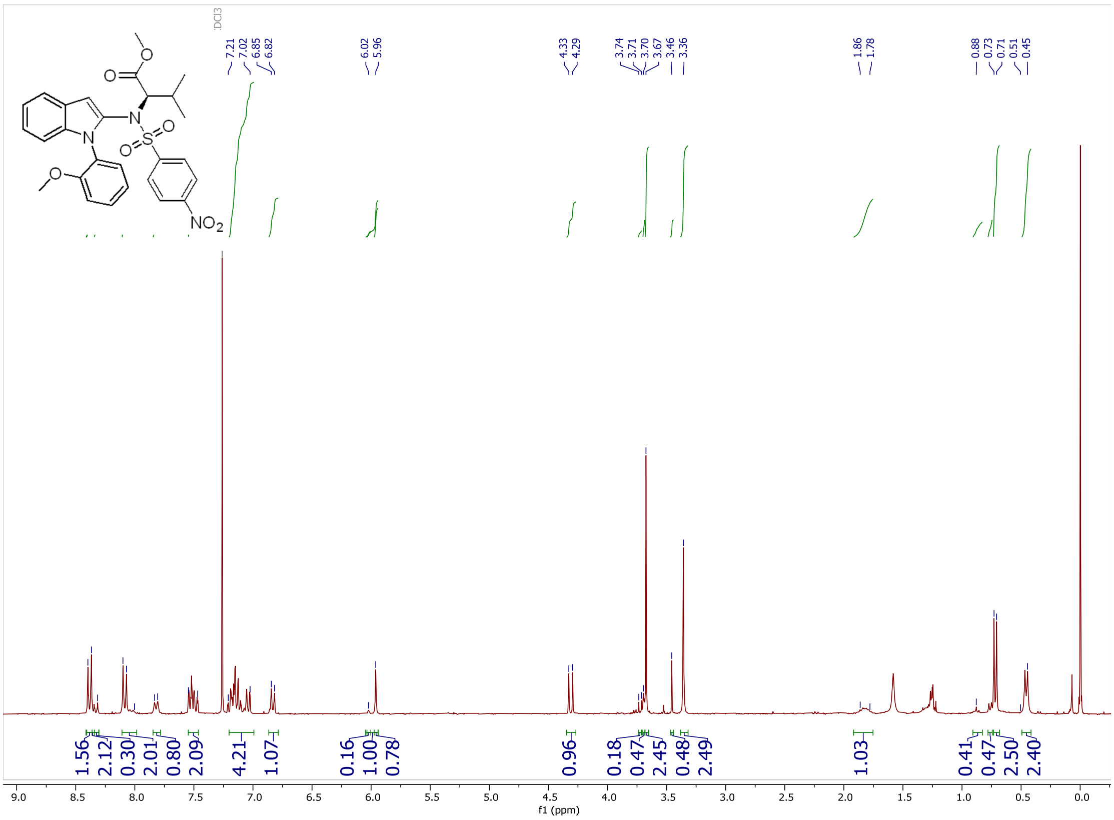

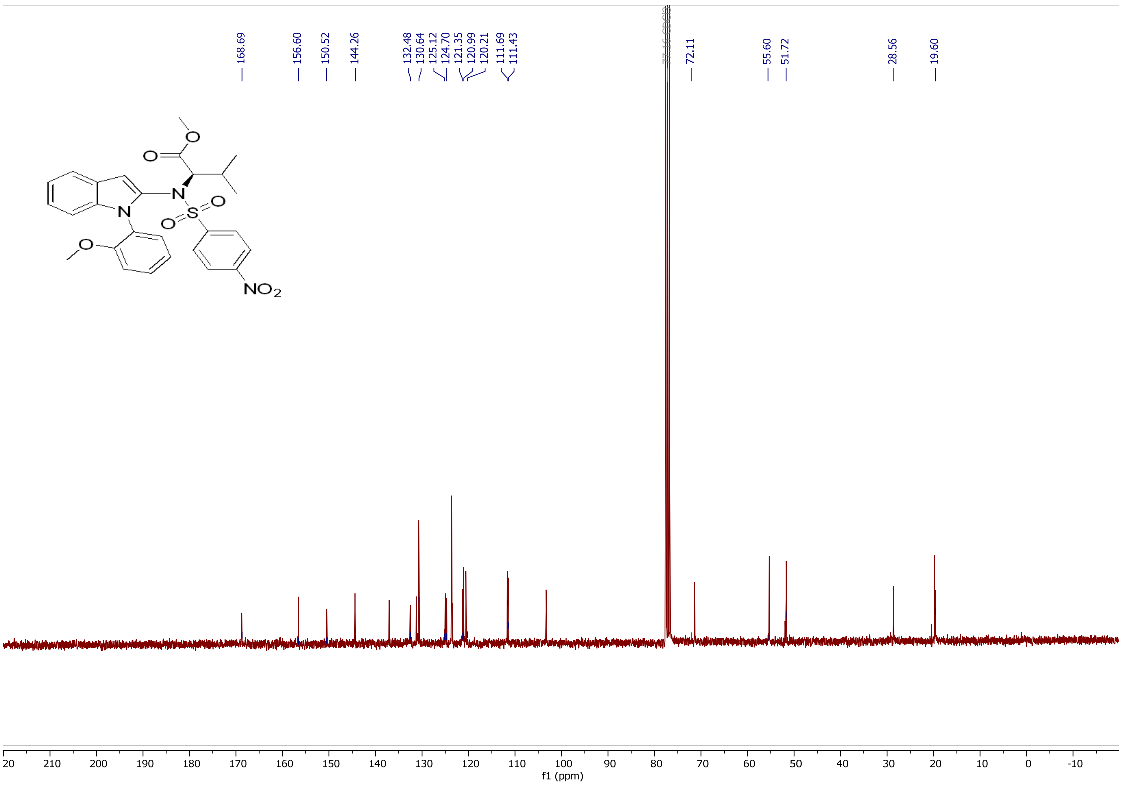

^{1}H NMR and ^{13}C NMR of **3o**

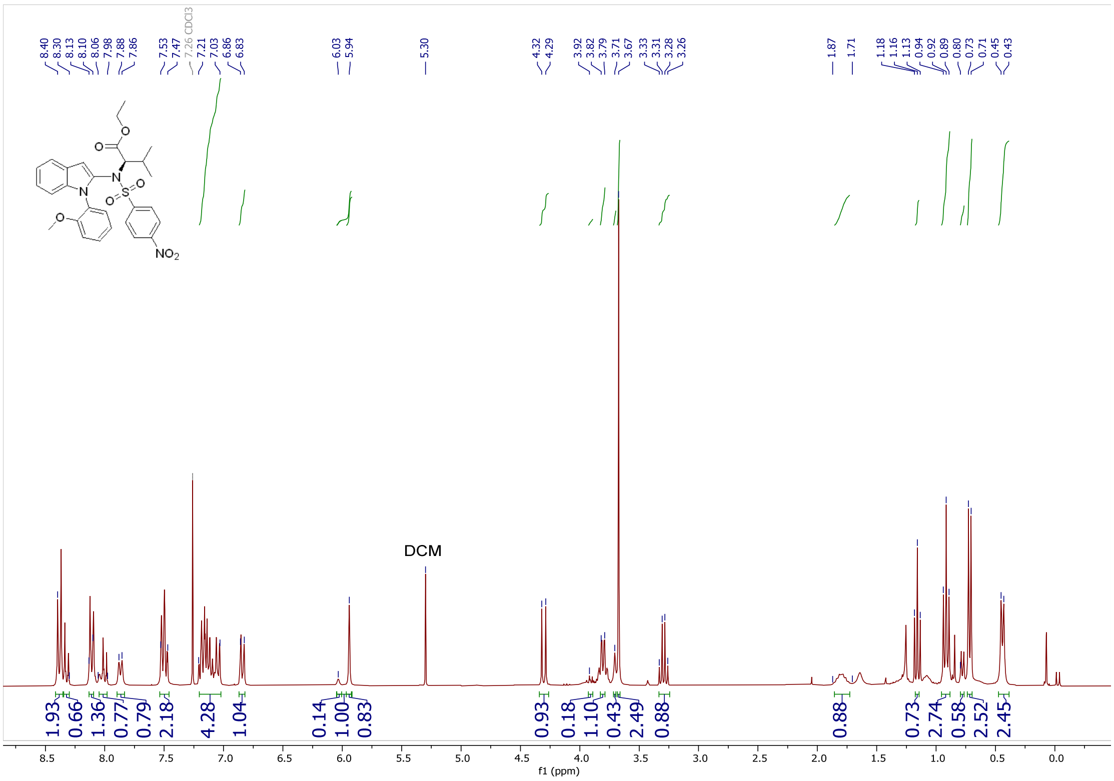

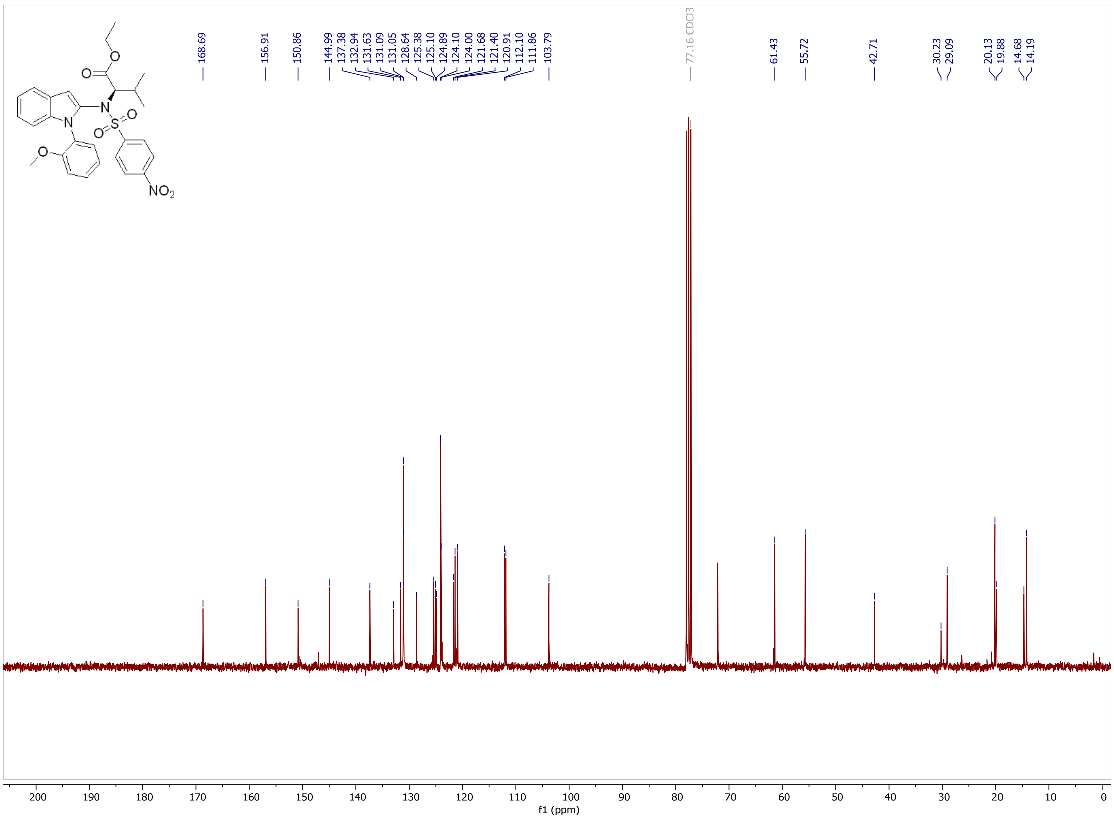

^{1}H NMR and ^{13}C NMR of **3p**

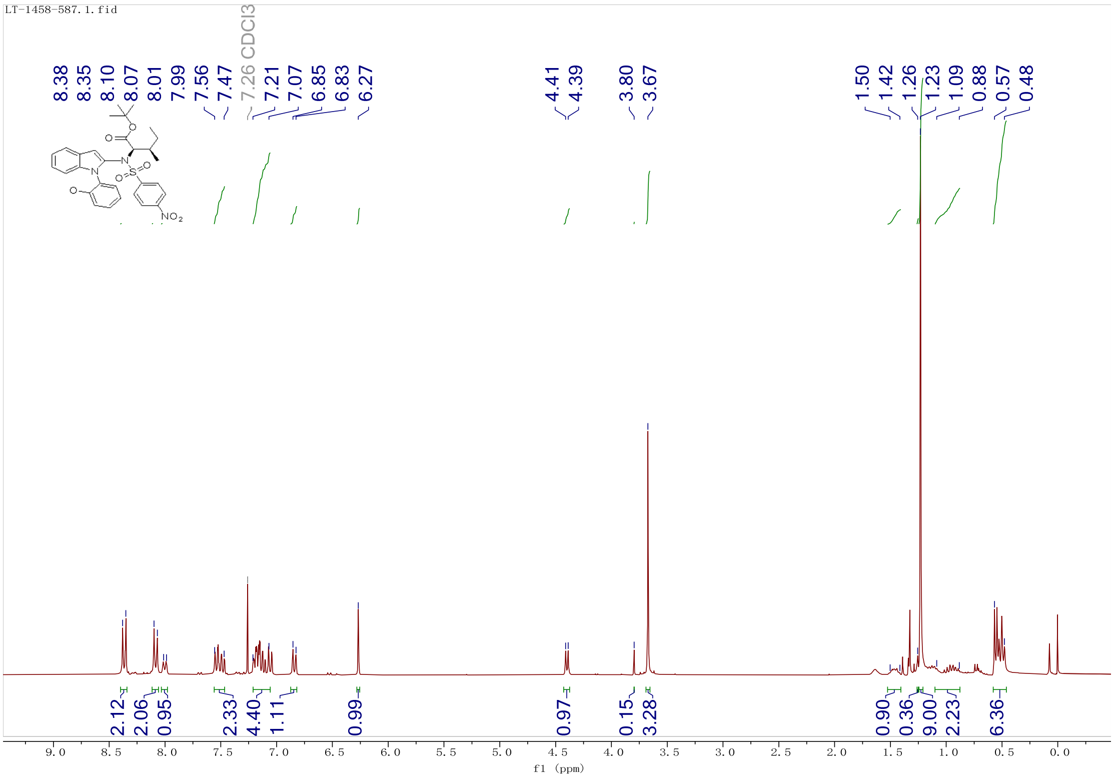

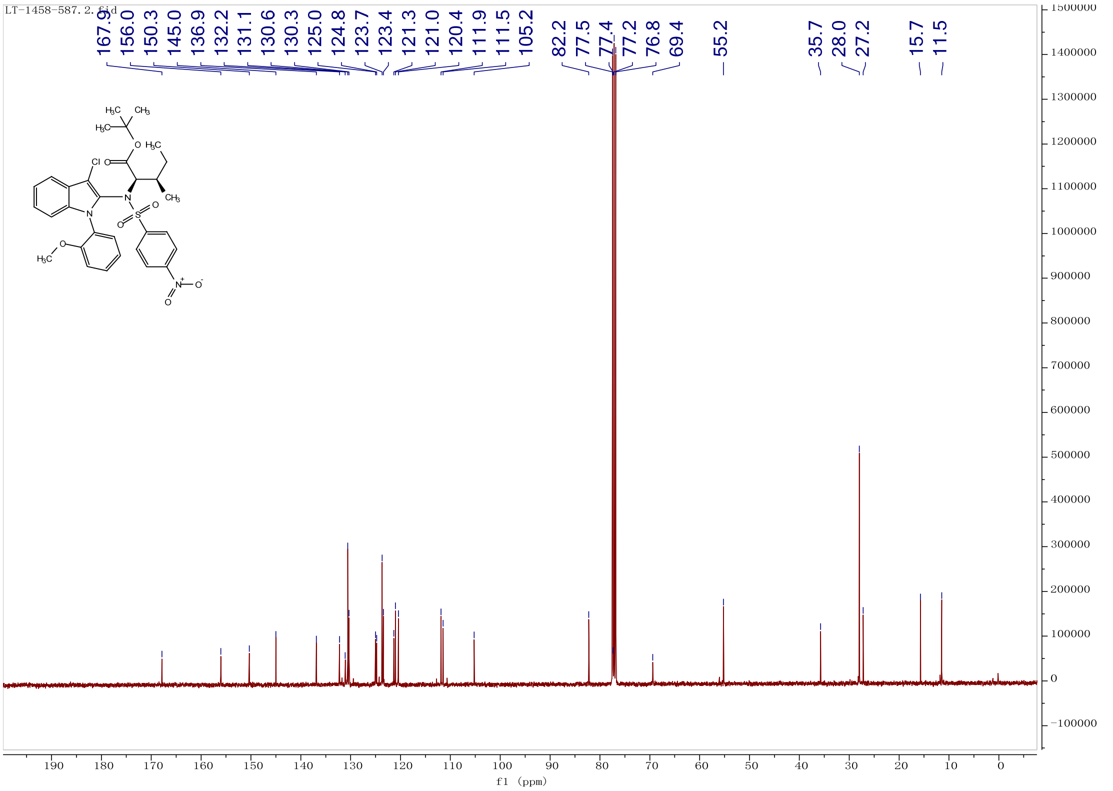

^{1}H NMR and ^{13}C NMR of **3q**

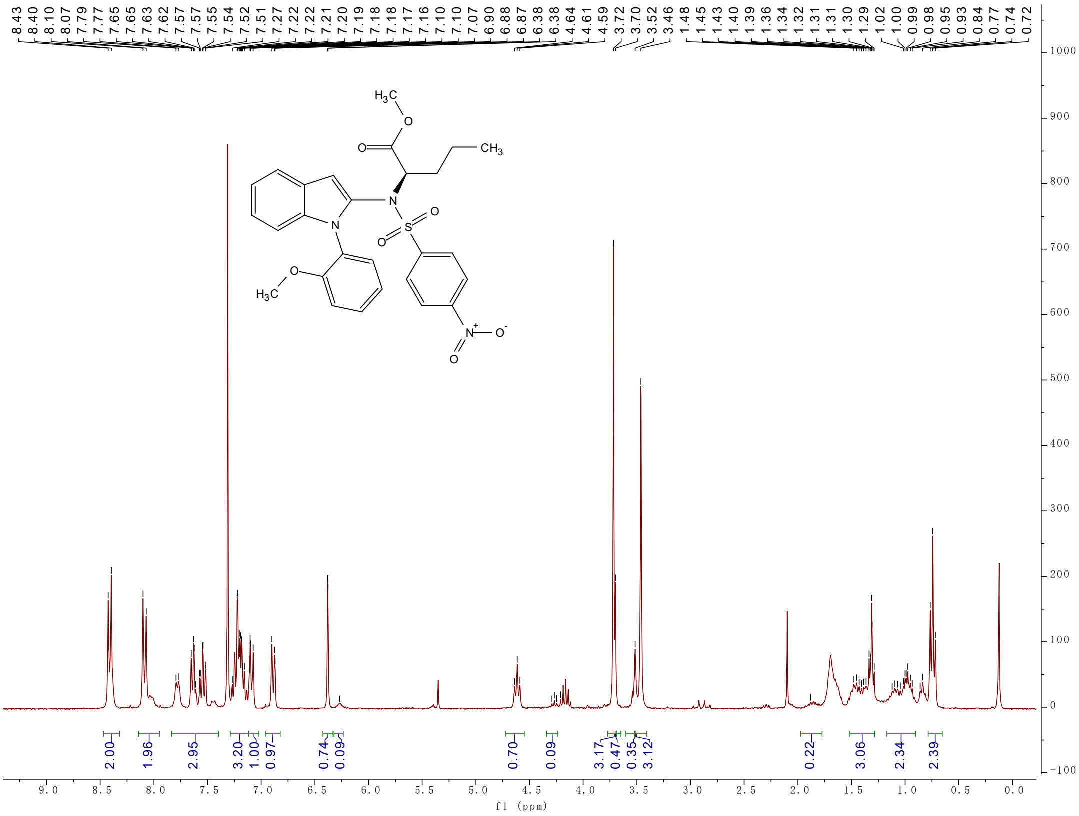

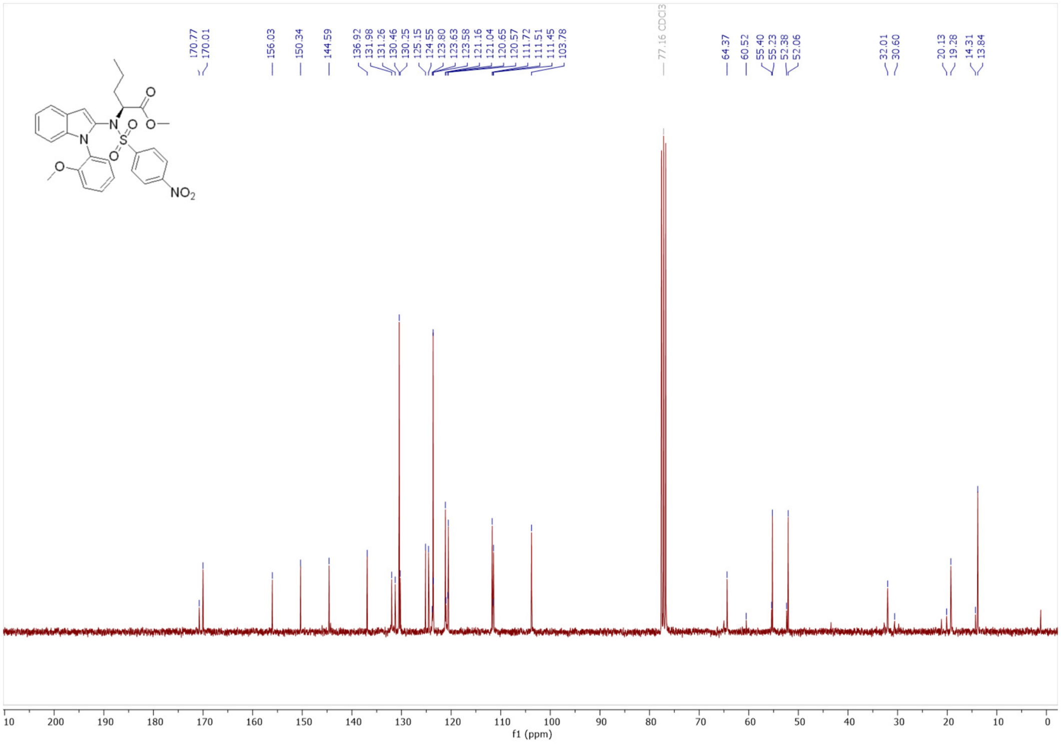

^{1}H NMR and ^{13}C NMR of **3r**

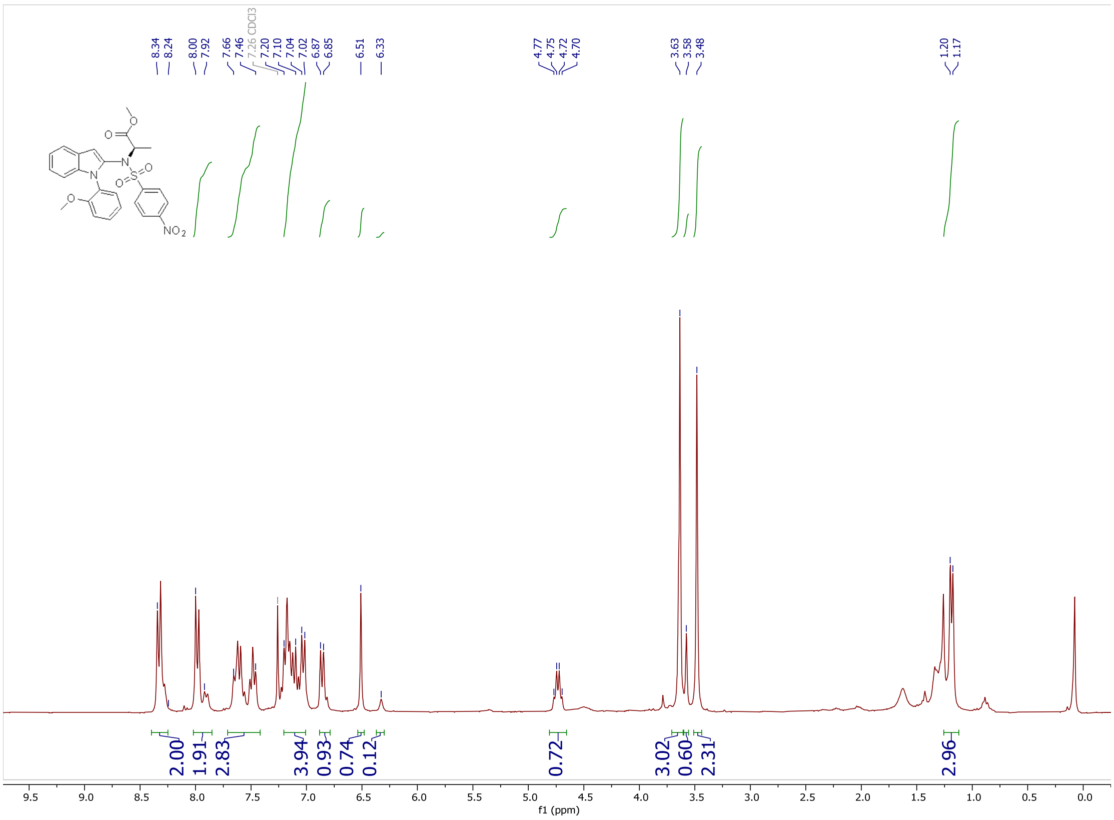

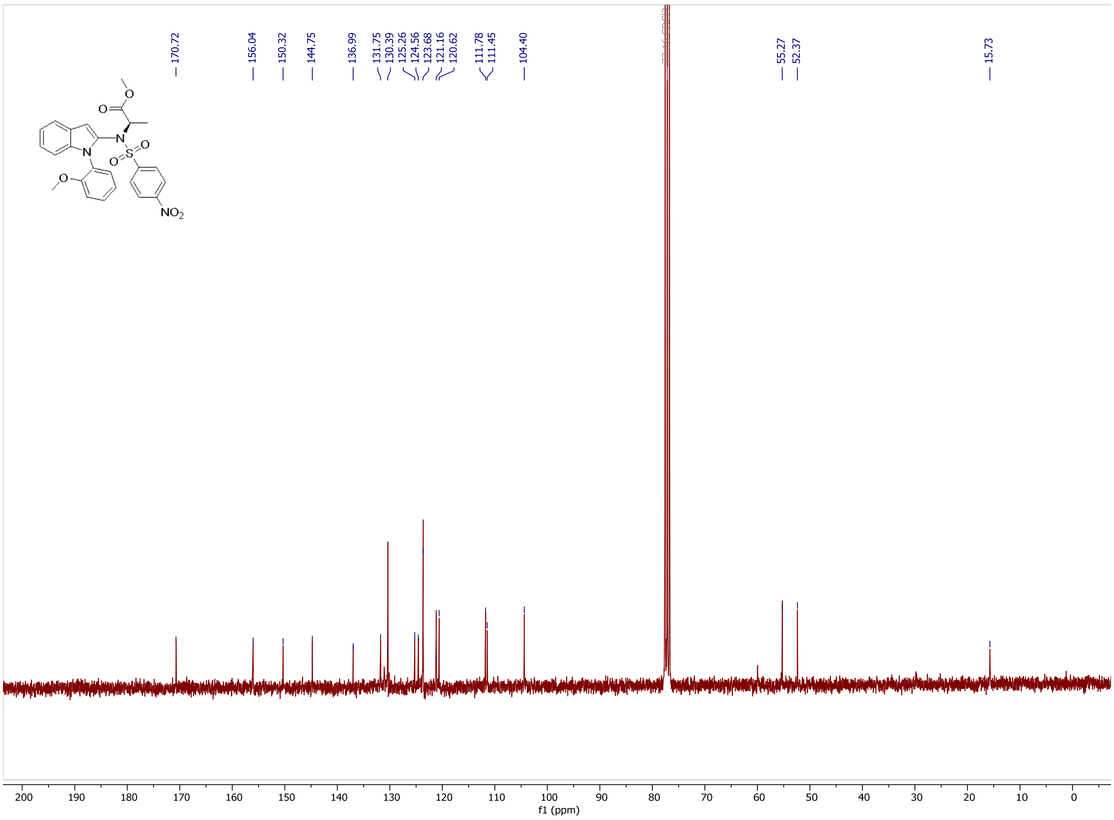

^{1}H NMR and ^{13}C NMR of **3s** and **4s**

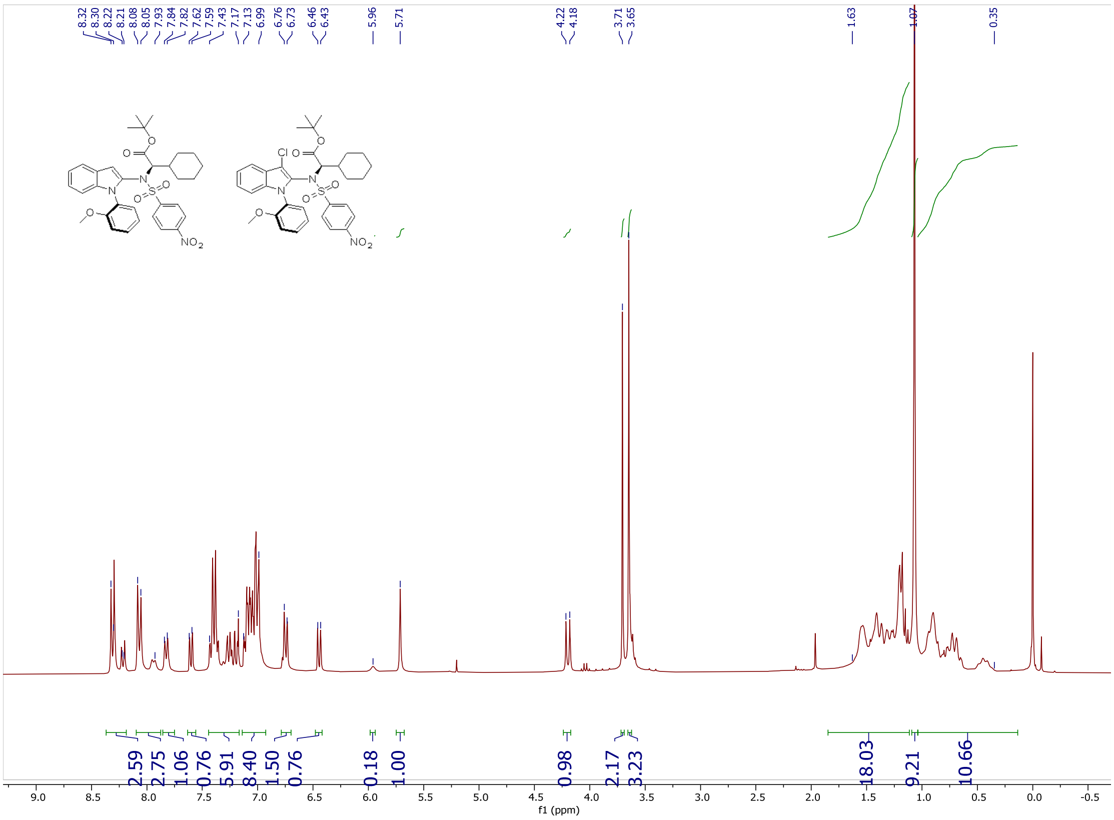

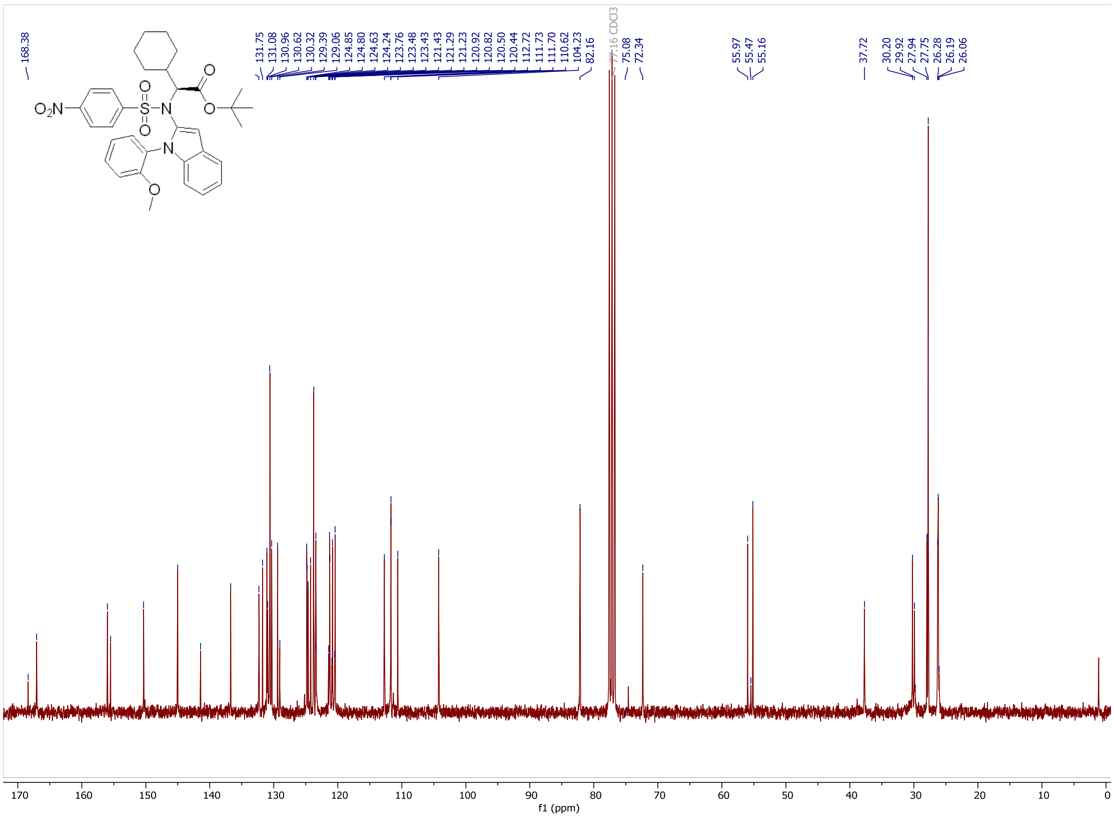

^{1}H NMR and ^{13}C NMR of **3t** and **4t**

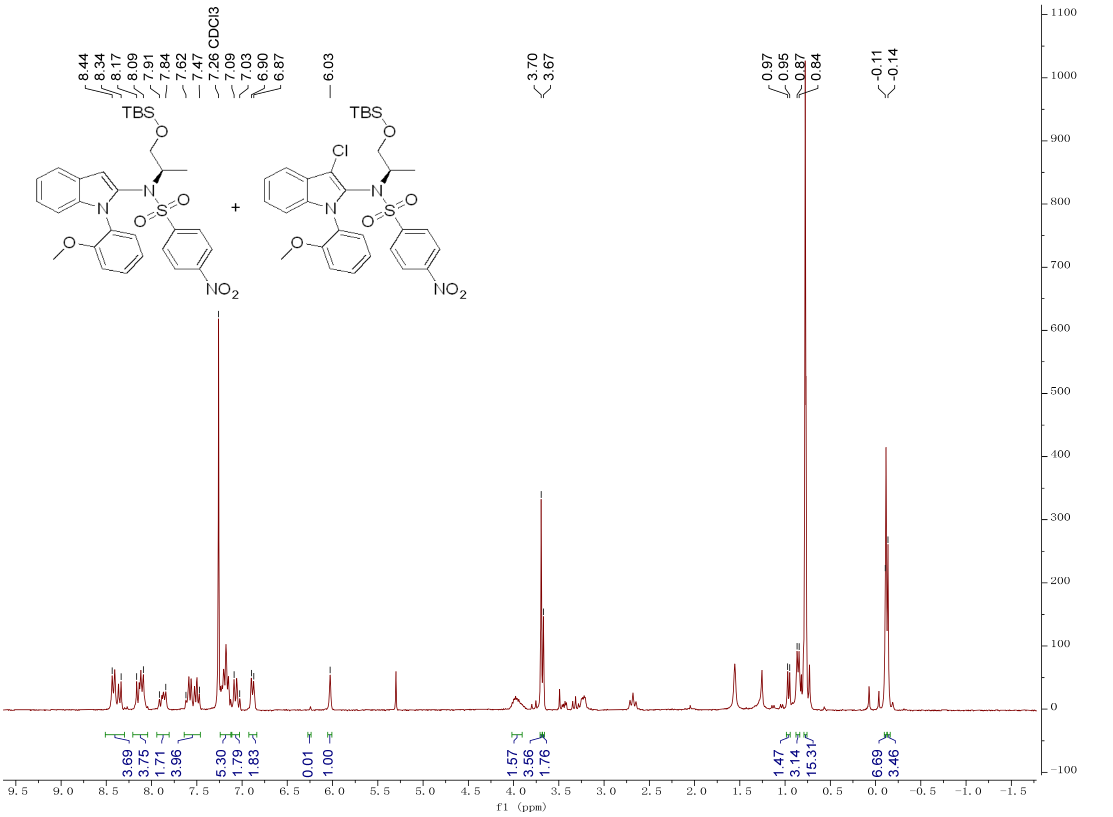

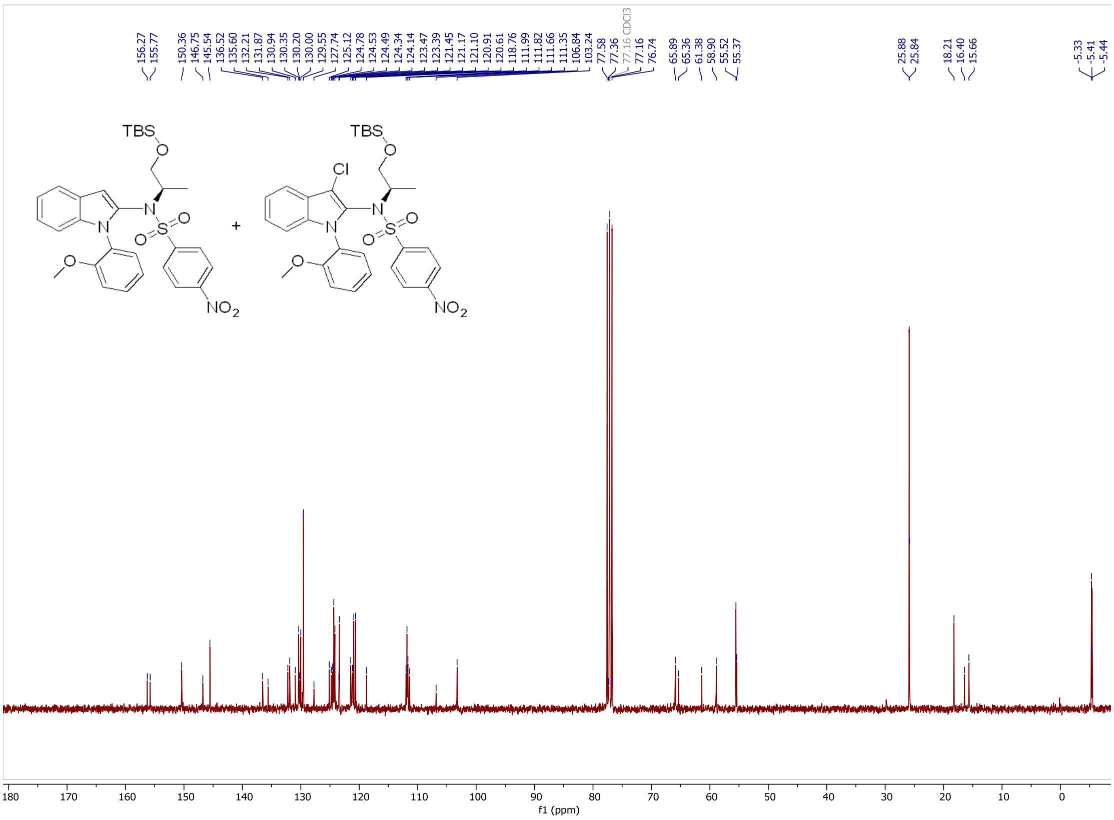

2. ORTEP drawings and X-ray crystallographic date of compound 2a

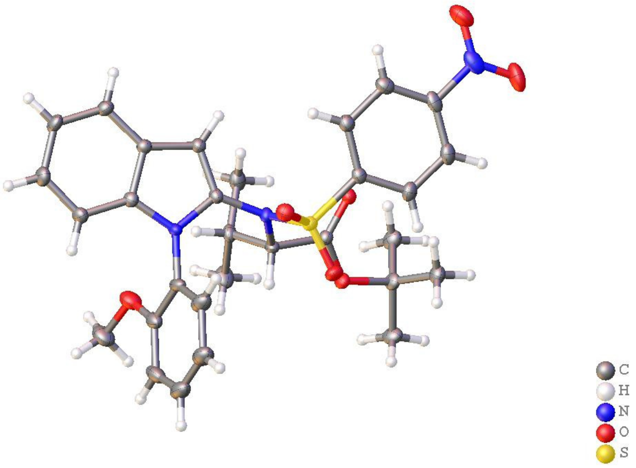

Single crystals of $C_{30}H_{33}N_3O_7S$ [**compound 3a**] were grown from a petroleum ether/CH2Cl2 solution of the compound at 25 ℃. A suitable crystal was selected on a Xcalibur,Atlas, Gemini ultra diffractometer. The crystal was kept at 293(2) K during data collection.Using Olex2[1], the structure was solved with the ShelXS[2] structure solution program usingDirect Methods and refined with the ShelXL[3] refinement package using Least Squaresminimisation.

Identification code	**Compound 3a**
Empirical formula	$C_{30}H_{33}N_3O_7S$
Formula weight	1159.30
Temperature/K	293(2)
Crystal system	orthorhombic
Space group	$P2_12_12_1$
a/Å	9.79670(10)
b/Å	10.76460(10)
c/Å	54.6347(3)
α/°	90
β/°	90
γ/°	90
Volume/Å^3	5761.64(9)
Z	4
ρ_{calc}g/cm^3	1.336
μ/mm^{-1}	1.435
F(000)	2448.0
Crystal size/mm^3	0.200 × 0.100 × 0.100
Radiation	CuKα (λ = 1.54178)
2Θ range for data collection/°	6.47 to 148.926
Index ranges	-12 ≤ h ≤ 11, -13 ≤ k ≤ 13, -67 ≤ l ≤ 68

Reflections collected	97339
Independent reflections	11701 [R_{int} = 0.0570, R_{sigma} = 0.0253]
Data/restraints/parameters	11701/0/739
Goodness-of-fit on F^2	1.009
Final R indexes [I>=2σ (I)]	R_1 = 0.0320, wR_2 = 0.0942
Final R indexes [all data]	R_1 = 0.0338, wR_2 = 0.0960
Largest diff. peak/hole / e Å^{-3}	0.66/-0.35
Flack parameter	-0.006(5)

[1]. Dolomanov, O.V., Bourhis, L.J., Gildea, R.J, Howard, J.A.K. & Puschmann, H. (2009), J. Appl. Cryst. 42, 339-341.
[2]. Sheldrick, G.M. (2015). Acta Cryst. A71, 3-8.
[3]. Sheldrick, G.M. (2015). Acta Cryst. C71, 3-8

3. **Stability experiments**

Product 3a was charged with DCM, the solution was stirred at 40-80 °C for 0-24 h. Dr value was determined by ^{1}H NMR. The *dr* value increased as time goes by at 40 °C and 60 °C and finally reach to around 6/1. At higher temperature, racemization was observed.

40℃	Time/ h	0 h	6 h	12 h	24 h
	dr	3.5/1	4.1/1	4.9/1	6.4/1
60℃	Time/ h	0 h	6 h	19 h	25 h
	dr	3.5/1	6.0/1	6/1	6.1/1
80℃	Time/ h	0 h	6 h	12 h	24h
	dr	3.5/1	5.9/1	5.1/1	4.6/1

4. **DFT calculations**

S1	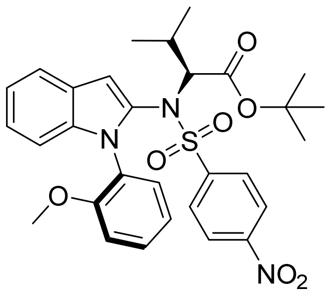	
	Geom.opt.	Single-point
level	M06-2X/def2SVP	M06-2X/def2TZVP
energy	Thermal correction to Gibbs Free Energy =0.444793 hartree	E = -2134.067331 hartree

cartesian coordinates of stationary point structure [Å]	C	0.09586700	-1.96563000	-4.60833100
	C	-0.99048500	-2.62215600	-3.93582200
	C	-1.86447800	-1.92528400	-3.07662800
	C	-1.63721800	-0.57270000	-2.90222700
	C	-0.56862000	0.09227200	-3.56057700
	C	0.29208300	-0.58545900	-4.40549100
	C	0.77873700	-2.95356700	-5.39489500
	C	0.12529000	-4.16541000	-5.21103700
	H	-2.67862400	-2.44304900	-2.57654700
	H	-2.28693800	0.00764100	-2.24677200
	H	-0.43646800	1.15948900	-3.38779500
	H	1.10981800	-0.07620300	-4.91154700
	H	1.62932700	-2.77807700	-6.02309600
	N	-0.97786600	-3.97873300	-4.32593400
	C	-1.83989000	-4.98727600	-3.78555700
	C	-2.87366900	-5.52704700	-4.56484200
	C	-1.67196400	-5.44728300	-2.45498300
	C	-3.72272300	-6.49522400	-4.02587300
	H	-3.00832600	-5.19134200	-5.59577100
	C	-2.53592800	-6.40794800	-1.90896800
	C	-3.55457800	-6.93032600	-2.70588000
	H	-4.51774400	-6.91630100	-4.64041400
	H	-2.40105700	-6.75341400	-0.88615800
	H	-4.22576100	-7.68704900	-2.29494600
	O	-0.64462400	-4.88913500	-1.74030600
	N	0.41381500	-5.41717300	-5.81343800
	C	1.30043600	-6.36818600	-5.09398700
	H	2.04869000	-6.78520100	-5.82840000
	C	2.04180800	-5.73190900	-3.92653800
	H	2.72747600	-6.44666200	-3.45197200
	H	2.64219400	-4.86906500	-4.24715000
	H	1.35277300	-5.37349100	-3.14189300
	C	0.45212600	-7.54755900	-4.55429900
	O	-0.06621100	-7.65885700	-3.46882400
	O	0.42625600	-8.51193400	-5.50988500
	C	-0.48953700	-9.70432300	-5.40503300
	C	-0.10594600	-10.50135900	-4.16978900
	C	-1.90662800	-9.16048700	-5.37618600
	C	-0.15009600	-10.42663400	-6.70390200
	H	0.96244100	-10.74592500	-4.15206200
	H	-0.32290700	-9.94646600	-3.24495000
	H	-0.66062400	-11.44615600	-4.11880800
	H	-2.04518700	-8.37259600	-6.13850100
	H	-2.65067700	-9.94001700	-5.56515800

	H	-2.15018300	-8.69516800	-4.41022800
	H	-0.81943500	-11.27932000	-6.87094900
	H	-0.25329800	-9.75309600	-7.57209100
	H	0.87830900	-10.80779400	-6.70963900
	S	-0.17863500	-5.69541600	-7.41392200
	O	-1.25696700	-6.65773800	-7.33480400
	O	-0.50128800	-4.39195500	-7.96143700
	C	1.19810700	-6.37297200	-8.21424100
	C	1.28890400	-7.75667200	-8.37991800
	C	2.19960900	-5.52792000	-8.70346900
	C	2.39344100	-8.31604400	-9.02861400
	H	0.49444600	-8.42298500	-7.98812900
	C	3.31005000	-6.06949100	-9.35802900
	H	2.12736100	-4.43902700	-8.58270100
	C	3.39193800	-7.46006600	-9.50964100
	H	2.47479000	-9.40179200	-9.15642100
	H	4.10152200	-5.41765500	-9.74613400
	N	4.57073600	-8.04086500	-10.20171200
	O	5.38596000	-7.27283200	-10.69044400
	O	4.66957300	-9.25751100	-10.24906500
	C	-0.50894902	-5.48925522	-0.44943497
	H	0.35213954	-5.08705073	0.04215229
	H	-1.38182675	-5.28209915	0.13372096
	H	-0.39564086	-6.54765664	-0.55828207

S2	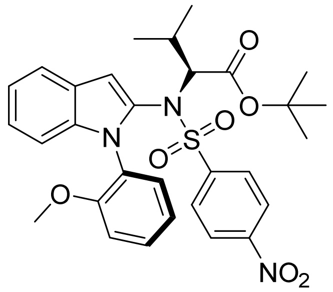			
	Geom.opt.		Single-point	
level	M06-2X/def2SVP		M06-2X/def2TZVP	
energy	Thermal correction to Gibbs Free Energy =0.446392 hartree		E = -2134.065257 hartree	
cartesian coordinates of stationary point structure [Å]	C	0.22113000	-2.02517100	-4.43731900
	C	-0.84314100	-2.72938500	-3.77945800
	C	-1.68498700	-2.09847900	-2.84026600
	C	-1.44431000	-0.76449300	-2.56926500
	C	-0.39396400	-0.05355600	-3.20945100
	C	0.43392200	-0.66596000	-4.13296000

	C	0.87054100	-2.95180600	-5.32001400
	C	0.22462300	-4.17612000	-5.20175200
	H	-2.48649400	-2.65067900	-2.35663300
	H	-2.06888400	-0.23442300	-1.84975000
	H	-0.24986800	0.99647100	-2.95827800
	H	1.23845200	-0.12215900	-4.62413800
	H	1.70154800	-2.73009800	-5.95990700
	N	-0.82643000	-4.06311900	-4.24211200
	C	-1.81036500	-5.03947100	-3.87640000
	C	-1.57641200	-5.90093900	-2.79412800
	C	-3.05298600	-5.11631000	-4.55410800
	C	-2.55394800	-6.81817500	-2.40330200
	H	-0.61623900	-5.85967400	-2.26955700
	C	-4.02594000	-6.05281400	-4.17507400
	C	-3.76792400	-6.89755500	-3.09551600
	H	-2.36460000	-7.47866900	-1.55765800
	H	-4.97025200	-6.11445800	-4.71267700
	H	-4.52170800	-7.62585600	-2.78884300
	O	-3.25369600	-4.21755000	-5.56415600
	H	-4.10898700	-4.38419000	-6.05213400
	N	0.51816400	-5.38726100	-5.87950500
	C	1.60052900	-6.25369000	-5.34634000
	H	2.15928600	-6.71051100	-6.21373700
	C	2.59493400	-5.52628200	-4.45363500
	H	3.39524300	-6.20158500	-4.11897000
	H	3.07720300	-4.68999900	-4.97976900
	H	2.12610400	-5.11013100	-3.54920000
	C	0.94594700	-7.40092500	-4.53570500
	O	0.64803900	-7.40706400	-3.36394500
	O	0.78319000	-8.44824000	-5.37980500
	C	-0.05292000	-9.65220300	-5.03251100
	C	0.51886500	-10.30504800	-3.78698400
	C	-1.47604100	-9.14866100	-4.87370000
	C	0.14844300	-10.49085300	-6.28970600
	H	1.58322000	-10.54394500	-3.89693000
	H	0.42798100	-9.65144500	-2.90649100
	H	-0.00484500	-11.23992200	-3.55216100
	H	-1.73363800	-8.41916200	-5.66246200
	H	-2.20758600	-9.96137800	-4.92465100
	H	-1.62584500	-8.63052900	-3.91696800
	H	-0.45266500	-11.40779900	-6.25570800
	H	-0.15125400	-9.93402300	-7.19307800
	H	1.19594600	-10.78751800	-6.42479400
	S	-0.33471300	-5.74073400	-7.34200400

	O	-1.36937500	-6.70642500	-7.03353300
	O	-0.74907100	-4.46528400	-7.89127200
	C	0.88111600	-6.46225100	-8.34194600
	C	1.00047600	-7.85355600	-8.39327200
	C	1.71526600	-5.64569500	-9.11193700
	C	1.97097400	-8.44548300	-9.20611400
	H	0.33911200	-8.49963700	-7.78322300
	C	2.69074400	-6.22094800	-9.93243200
	H	1.61550300	-4.55240600	-9.08379900
	C	2.80624200	-7.61653600	-9.96570300
	H	2.07600400	-9.53597800	-9.24708300
	H	3.35191300	-5.59067800	-10.53891600
	N	3.84304400	-8.23307900	-10.83247000
	O	4.56851500	-7.49005100	-11.47659800
	O	3.92192800	-9.45167100	-10.85923800

S3	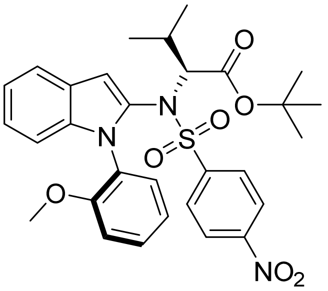			
	Geom.opt.		Single-point	
level	M06-2X/def2SVP		M06-2X/def2TZVP	
energy	Thermal correction to Gibbs Free Energy =0.4462hartree		E = -2134.066547 hartree	
cartesian coordinates of stationary point structure [Å]	C	-0.40912300	-3.03947800	-6.12461200
	C	-1.16270200	-3.57995900	-5.05145600
	C	-2.16585900	-2.85376600	-4.39733000
	C	-2.41288600	-1.56459000	-4.84622900
	C	-1.68505400	-1.00965900	-5.92134400
	C	-0.68922000	-1.73095600	-6.56145200
	C	0.49293900	-4.06511700	-6.55761100
	C	0.27981400	-5.14660600	-5.74493700
	H	-2.72783700	-3.29025100	-3.57045000
	H	-3.18599900	-0.96823900	-4.35950200
	H	-1.91226900	0.00556900	-6.24968700
	H	-0.12923600	-1.29575300	-7.39090400
	H	1.20309300	-4.03274600	-7.37920500
	N	-0.72588800	-4.87126000	-4.83014200
	C	-1.12336200	-5.69279000	-3.74486800

	C	-0.23810900	-5.86627800	-2.66826700
	C	-2.38003300	-6.29514000	-3.72893000
	C	-0.61222700	-6.69214900	-1.60566700
	C	-2.75884700	-7.09976800	-2.65615500
	C	-1.86569700	-7.30200400	-1.60336000
	H	0.07989600	-6.84167800	-0.77428300
	H	-3.73962700	-7.57467200	-2.64807500
	H	-2.14545500	-7.93947100	-0.76356500
	N	0.89943500	-6.41256700	-5.75043600
	C	0.19466400	-7.62906800	-6.24223700
	H	0.83534800	-8.06985400	-7.02428700
	C	-0.09138800	-8.65744200	-5.15842500
	H	0.83726800	-9.00129300	-4.68837200
	H	-0.75423800	-8.24643500	-4.38746600
	H	-0.58978200	-9.52399700	-5.61215300
	C	-1.09325300	-7.24406200	-6.98122600
	O	-2.18279400	-7.61460000	-6.62779800
	O	-0.82770500	-6.50480900	-8.04119700
	C	-1.88921200	-5.93009600	-8.86805000
	C	-2.60026300	-7.05404200	-9.61298200
	C	-2.84625400	-5.10562500	-8.01178900
	C	-1.12866500	-5.02923400	-9.83050200
	H	-1.87759300	-7.63506400	-10.20343100
	H	-3.11719400	-7.72251000	-8.91383900
	H	-3.34104100	-6.62106200	-10.29956200
	H	-2.27979000	-4.39913700	-7.38692500
	H	-3.50181400	-4.52583000	-8.67672500
	H	-3.46840400	-5.74086800	-7.37116200
	H	-1.82547600	-4.58421000	-10.55346900
	H	-0.62787400	-4.22237600	-9.27703000
	H	-0.37139500	-5.60809000	-10.37697600
	S	2.56618200	-6.39460900	-6.02274600
	O	2.83553000	-6.70349900	-7.41957700
	O	3.09774600	-5.18311000	-5.43237800
	C	3.11623300	-7.78114900	-5.05050600
	C	3.64655600	-8.89235600	-5.69655400
	C	3.01463900	-7.68934300	-3.66222700
	C	4.07986100	-9.96914700	-4.92417100
	H	3.71418200	-8.91256400	-6.78441400
	C	3.44562000	-8.76065600	-2.89036100
	H	2.57968700	-6.79716500	-3.21002600
	C	3.96579000	-9.87596000	-3.54334000
	H	4.49882000	-10.86589500	-5.37674500
	H	3.38346300	-8.74752700	-1.80391300

	N	4.42002800	-11.02047900	-2.72253800
	O	4.31981900	-10.92034800	-1.52416200
	O	4.86170400	-11.98180700	-3.30241300
	H	-3.03972000	-6.14207000	-4.58397000
	O	0.94748900	-5.22076800	-2.72105200
	C	1.58541854	-5.25783959	-1.44176642
	H	2.54790388	-4.79532874	-1.50963160
	H	0.98603441	-4.73105937	-0.72892613
	H	1.69964902	-6.27486957	-1.12951425

S4	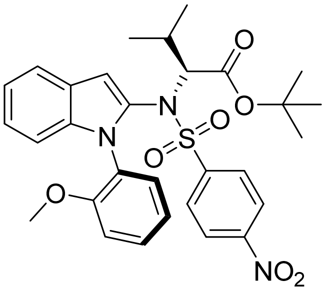			
	Geom.opt.		Single-point	
level	M06-2X/def2SVP		M06-2X/def2TZVP	
energy	Thermal correction to Gibbs Free Energy =0.447312hartree		E = -2134.071547 hartree	
cartesian coordinates of stationary point structure [Å]	C	-0.90417300	-2.78559300	0.65098200
	C	-2.18571400	-2.44798800	0.12522800
	C	-3.09870500	-4.42735500	0.67241200
	C	-1.88453000	-4.88611100	1.21827200
	C	-0.76462700	-4.06115500	1.21276800
	C	-0.06041800	-1.64663800	0.45777300
	C	-0.82149100	-0.68664300	-0.16438600
	H	-3.97196600	-5.07595300	0.68244000
	H	-1.83611600	-5.88387100	1.64160800
	H	0.18151000	-4.39143400	1.63163400
	H	0.97396300	-1.54479900	0.74344000
	N	-2.11512800	-1.16861100	-0.39021400
	C	-3.22924400	-0.40621500	-0.84221500
	C	-4.04331400	0.22281000	0.11826900
	C	-3.51095400	-0.29183100	-2.19771000
	C	-5.16503700	0.94201300	-0.30472200
	C	-4.61961900	0.44437300	-2.62091700
	C	-5.44212700	1.04752900	-1.67052400
	H	-5.81122600	1.43032700	0.41419300
	H	-4.83896400	0.53894300	-3.67927100
	H	-6.31167900	1.61594200	-1.98697300

	N	-0.49119900	0.62694500	-0.55915000
	C	-0.51289200	1.68287100	0.50226400
	H	-1.25317500	1.29604700	1.20629900
	C	-0.94042700	3.09345100	0.06204600
	H	-0.13703300	3.51275400	-0.54633900
	C	0.84912000	1.71646900	1.21157700
	O	1.77244000	2.42356900	0.85129100
	O	0.86701100	0.85169200	2.22486600
	C	2.07431700	0.63098800	3.06095800
	C	3.23358800	0.14318400	2.19282000
	C	2.40715000	1.92224400	3.80684800
	C	1.60893800	-0.46061900	4.02145200
	H	2.94468200	-0.74105900	1.61583000
	H	3.56897900	0.91828800	1.50474500
	H	4.07000300	-0.13759200	2.84085100
	H	2.72276600	2.70634500	3.11681800
	H	1.53549000	2.27120400	4.36979700
	H	3.21940400	1.73064700	4.51539000
	H	1.31401300	-1.35903200	3.47044400
	H	2.42096300	-0.72168100	4.70661800
	H	0.75277300	-0.11706800	4.61014500
	S	0.34936200	0.75434300	-2.02506200
	O	-0.25278300	-0.24977700	-2.90753400
	O	0.38259000	2.16603500	-2.40802400
	C	2.03963100	0.22864200	-1.73233300
	C	3.02023400	1.19413700	-1.50380100
	C	2.33262000	-1.13836900	-1.74433100
	C	4.32716000	0.78350800	-1.26023300
	H	2.76250700	2.24405300	-1.50670500
	C	3.63574400	-1.55143700	-1.48917600
	H	1.55723700	-1.86372300	-1.95396700
	C	4.60605700	-0.58070600	-1.24671200
	H	5.11567400	1.50116800	-1.07719000
	H	3.90148900	-2.60004500	-1.48219000
	N	5.98144600	-1.01518400	-0.96603900
	O	6.21141800	-2.22448300	-0.94812400
	O	6.82965000	-0.14704100	-0.76058400
	H	-2.84487500	-0.77160100	-2.90548300
	O	-3.63981800	0.09406200	1.41054400
	C	-4.40806200	0.74807000	2.42021600
	H	-5.43340400	0.36320000	2.44683900
	H	-3.90766700	0.52405800	3.36229200
	H	-4.42578400	1.83218200	2.26119300
	H	-1.84792832	3.09272322	-0.50482331

	H	-1.05217990	3.69674561	0.93865703
	C	-3.27366400	-3.21892900	0.12307500
	H	-4.18088969	-2.84045250	-0.29953249
TS 1	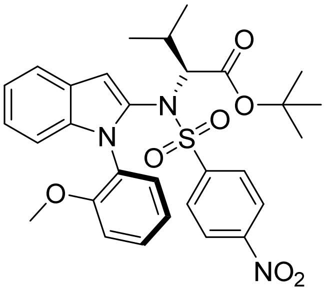			
	Geom.opt.		Single-point	
level	M06-2X/def2SVP		M06-2X/def2TZVP	
energy	Thermal correction to Gibbs Free Energy =0.447657hartree		E = -2134.026249 hartree	
cartesian coordinates of stationary point structure [Å]	C	1.14102900	-3.22317800	-5.66201700
	C	-0.26533500	-3.02591500	-5.64365600
	C	-0.76617000	-1.82513500	-6.18335600
	C	0.12500400	-0.85816600	-6.63054200
	C	1.51839200	-1.03843000	-6.58258000
	C	2.03141600	-2.23025400	-6.10590400
	C	1.38526900	-4.60369900	-5.40737200
	C	0.17380000	-5.20224600	-5.25462600
	H	-1.82579300	-1.65686700	-6.30392800
	H	-0.28150000	0.05847500	-7.06087700
	H	2.18354800	-0.25937700	-6.95757500
	H	3.10392900	-2.43305600	-6.11602600
	H	2.33235400	-5.12862600	-5.51320200
	N	-0.87940600	-4.25358200	-5.27018800
	C	-2.14523700	-4.51267000	-4.61138200
	C	-2.35357900	-5.75776000	-3.98590500
	C	-3.21028700	-3.58802500	-4.48591400
	C	-3.52918300	-6.10171800	-3.33034200
	H	-1.59679800	-6.52606200	-4.03715500
	C	-4.39441200	-3.94524500	-3.82674600
	C	-4.57542000	-5.19074700	-3.24879700
	H	-3.60829300	-7.09600000	-2.89018900
	H	-5.18402400	-3.19173100	-3.77354200
	H	-5.51033800	-5.43486100	-2.74393200
	O	-3.11742900	-2.33251700	-4.97260200
	N	-0.04274800	-6.58598100	-5.40933600
	C	0.79500500	-7.57592500	-4.72171700
	H	1.69928300	-7.81429700	-5.30352500

	C	1.18699300	-7.11452400	-3.31738200
	H	1.69949000	-7.94515200	-2.81455100
	H	1.86308500	-6.25396400	-3.34855100
	H	0.29419100	-6.84407500	-2.73678400
	C	-0.00984000	-8.86692200	-4.56059000
	O	-1.20041600	-8.88144500	-4.37868400
	O	0.79663900	-9.91174200	-4.57068700
	C	0.29425100	-11.27633800	-4.41809900
	C	-0.35055300	-11.44033200	-3.04618900
	C	-0.66867900	-11.59018900	-5.55801900
	C	1.55462800	-12.12274800	-4.52690500
	H	0.35539700	-11.14035700	-2.25814800
	H	-1.26345700	-10.83980900	-2.96142800
	H	-0.60676800	-12.49817600	-2.89444800
	H	-0.19119300	-11.36959900	-6.52299900
	H	-0.92548700	-12.65827500	-5.52825600
	H	-1.58872400	-11.00049500	-5.47515000
	H	1.30011900	-13.18613200	-4.42463900
	H	2.03550600	-11.96631500	-5.50233700
	H	2.26642900	-11.85194700	-3.73473000
	S	-0.63349100	-7.03126800	-6.94443400
	O	-0.09148900	-8.35849900	-7.20400400
	O	-2.06129800	-6.79165400	-6.99416800
	C	0.15229600	-5.83697600	-8.01212700
	C	1.50291600	-6.00280800	-8.32073600
	C	-0.55902800	-4.68989000	-8.35019700
	C	2.18192300	-4.94975100	-8.92296300
	H	2.02119600	-6.92654700	-8.05899200
	C	0.11520300	-3.63744400	-8.96507000
	H	-1.61493900	-4.60675600	-8.09082900
	C	1.47554300	-3.78180100	-9.20042300
	H	3.24409700	-5.01009500	-9.15239700
	H	-0.38765500	-2.70571600	-9.21728600
	N	2.21799500	-2.62287100	-9.73778600
	O	1.58128900	-1.64841600	-10.05587900
	O	3.41939900	-2.71788500	-9.81269500
	C	-4.29590041	-1.59834519	-4.63040776
	H	-5.14891342	-2.06439135	-5.07769936
	H	-4.41258431	-1.58665583	-3.56685323
	H	-4.20799639	-0.59464324	-4.99062336

Transition State 2	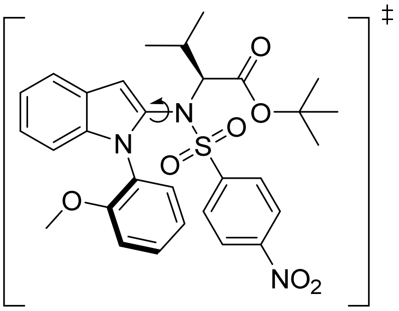	
	Geom.opt.	Single-point
level	M06-2X/def2SVP	M06-2X/def2TZVP
energy	Thermal correction to Gibbs Free Energy =0.449464hartree	E =-2134.034497 hartree

cartesian coordinates of stationary point structure [Å]			
C	0.05785700	-2.41578200	-5.18301300
C	-1.15231200	-3.06133900	-4.86306500
C	-2.35474600	-2.36668200	-4.70690700
C	-2.33211700	-0.99421900	-4.92899900
C	-1.13923600	-0.33262700	-5.28071200
C	0.05603200	-1.02803500	-5.40640600
C	1.07496700	-3.42378200	-5.18003000
C	0.46775400	-4.62569200	-4.89421900
H	-3.27441800	-2.88460800	-4.43128300
H	-3.25529500	-0.42127300	-4.83061600
H	-1.15686000	0.74508900	-5.45012000
H	0.98018200	-0.50899500	-5.66588200
H	2.12276900	-3.27630700	-5.41286300
N	-0.90766800	-4.42718100	-4.71024700
C	-1.84082200	-5.15163800	-3.90561600
C	-1.77202800	-4.99883900	-2.50828100
C	-2.88892000	-5.86402200	-4.47357800
C	-2.73330400	-5.60744900	-1.70191100
C	-3.84685500	-6.48103200	-3.66883400
C	-3.76087700	-6.35014800	-2.28429500
H	-2.66994900	-5.49816800	-0.61745900
H	-4.65496300	-7.05367700	-4.12332400
H	-4.50461000	-6.82425800	-1.64228800
N	1.09521500	-5.89446600	-4.93114500
C	0.43739900	-7.16894300	-4.63772700
H	1.19299200	-7.93696600	-4.84085800
C	0.04368800	-7.37580600	-3.16787000
H	0.60505500	-8.21722100	-2.74307800
H	0.28640800	-6.48046700	-2.58441400
H	-1.02681600	-7.59414100	-3.07623600
C	-0.63601300	-7.57744800	-5.65722500

	O	-1.49052500	-8.38783800	-5.41405300
	O	-0.39163500	-7.02528100	-6.83641400
	C	-1.12017200	-7.39314900	-8.05189400
	C	-0.95036500	-8.88352000	-8.32951700
	C	-2.58954400	-7.01104800	-7.91973100
	C	-0.44854700	-6.55180100	-9.12780600
	H	0.11452600	-9.15408400	-8.36480100
	H	-1.44794700	-9.49181300	-7.56559000
	H	-1.39161000	-9.11468100	-9.30880700
	H	-2.68533100	-5.93279900	-7.72607000
	H	-3.10026400	-7.23153500	-8.86718900
	H	-3.07880200	-7.57961000	-7.11904300
	H	-0.94056100	-6.72537100	-10.09427900
	H	-0.52047000	-5.48582000	-8.87329200
	H	0.61611800	-6.80590400	-9.22101500
	S	2.49111200	-5.87527700	-5.93166600
	O	2.14792300	-5.45001000	-7.27673900
	O	3.52458200	-5.15005900	-5.20878800
	C	3.01169600	-7.57814400	-6.01034100
	C	2.65350900	-8.32635900	-7.12894200
	C	3.84167300	-8.07109000	-5.00438400
	C	3.11304600	-9.63599300	-7.23125200
	H	2.02677400	-7.87838200	-7.89981700
	C	4.30058300	-9.38086800	-5.10405800
	H	4.12414100	-7.43713400	-4.16342000
	C	3.92004200	-10.13006800	-6.21249400
	H	2.86109600	-10.26705700	-8.08175300
	H	4.94668100	-9.81957100	-4.34606700
	N	4.40815500	-11.52421300	-6.31801400
	O	5.09962800	-11.94013800	-5.42152700
	O	4.08157000	-12.15502700	-7.29287900
	H	-2.93757500	-5.93349800	-5.55865400
	O	-0.73901700	-4.28445100	-2.02291300
	C	-0.81712529	-4.19735275	-0.59770670
	H	0.00353679	-3.61732293	-0.23031160
	H	-1.73793615	-3.72907148	-0.31892360
	H	-0.77542116	-5.18049250	-0.17747225

Transition State 3	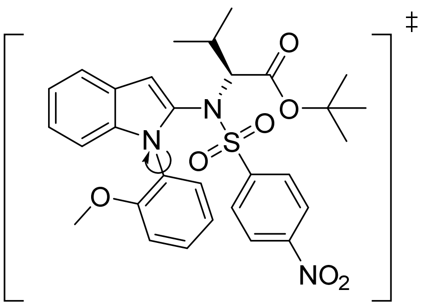<br>TS3	
	Geom.opt.	Single-point
level	M06-2X/def2SVP	M06-2X/def2TZVP
energy	Thermal correction to Gibbs Free Energy =0.447731hartree	E =-2134.02655 hartree

cartesian coordinates of stationary point structure [Å]			
C	1.14102900	-3.22317800	-5.66201700
C	-0.26533500	-3.02591500	-5.64365600
C	-0.76617000	-1.82513500	-6.18335600
C	0.12500400	-0.85816600	-6.63054200
C	1.51839200	-1.03843000	-6.58258000
C	2.03141600	-2.23025400	-6.10590400
C	1.38526900	-4.60369900	-5.40737200
C	0.17380000	-5.20224600	-5.25462600
H	-1.82579300	-1.65686700	-6.30392800
H	-0.28150000	0.05847500	-7.06087700
H	2.18354800	-0.25937700	-6.95757500
H	3.10392900	-2.43305600	-6.11602600
H	2.33235400	-5.12862600	-5.51320200
N	-0.87940600	-4.25358200	-5.27018800
C	-2.14523700	-4.51267000	-4.61138200
C	-2.35357900	-5.75776000	-3.98590500
C	-3.21028700	-3.58802500	-4.48591400
C	-3.52918300	-6.10171800	-3.33034200
H	-1.59679800	-6.52606200	-4.03715500
C	-4.39441200	-3.94524500	-3.82674600
C	-4.57542000	-5.19074700	-3.24879700
H	-3.60829300	-7.09600000	-2.89018900
H	-5.18402400	-3.19173100	-3.77354200
H	-5.51033800	-5.43486100	-2.74393200
O	-3.11742900	-2.33251700	-4.97260200
H	-3.91306900	-1.83684400	-4.74157100
N	-0.04274800	-6.58598100	-5.40933600
C	-0.69866825	-6.65256103	-6.72081411
H	-1.41875302	-7.48442844	-6.77096391
C	-1.39612743	-5.33904723	-7.07709272

	H	-1.75330870	-5.41086829	-8.11280423
	H	-2.25347741	-5.14642285	-6.42403662
	H	-0.69485778	-4.49661348	-7.00055276
	C	0.37023242	-6.89583746	-7.78790294
	O	1.48643859	-6.44919479	-7.71464978
	O	-0.13916469	-7.57718701	-8.79707911
	C	0.65991840	-7.93651310	-9.96761318
	C	1.11565461	-6.67395643	-10.69084100
	C	1.82793939	-8.81432616	-9.53135013
	C	-0.31886189	-8.72629727	-10.82495145
	H	0.24892363	-6.03972699	-10.92725048
	H	1.82684936	-6.10246470	-10.08354763
	H	1.60266312	-6.95711892	-11.63442305
	H	1.45818764	-9.66089704	-8.93600098
	H	2.33606072	-9.20647532	-10.42335666
	H	2.54895162	-8.24755686	-8.93134762
	H	0.17519333	-9.05477661	-11.74908860
	H	-0.67429619	-9.61161042	-10.27991053
	H	-1.18451619	-8.10314651	-11.08926345
	S	0.86018225	-7.95463197	-4.94533689
	O	-0.09700668	-8.86856874	-4.33608012
	O	1.68657845	-8.38021360	-6.05640429
	C	1.94117268	-7.28663730	-3.69273730
	C	1.41518105	-7.02192603	-2.42775710
	C	3.21828803	-6.88599188	-4.07292026
	C	2.16875709	-6.26335322	-1.53930487
	H	0.41750224	-7.37216090	-2.15870255
	C	3.98127088	-6.13635316	-3.18062110
	H	3.58796192	-7.11864671	-5.07209192
	C	3.42060231	-5.81366802	-1.95250061
	H	1.79309816	-5.99551584	-0.55358764
	H	4.97152004	-5.76890654	-3.44327245
	N	4.17601860	-4.91601148	-1.05435157
	O	5.29418977	-4.60480781	-1.38448145
	O	3.62154640	-4.53788244	-0.05056395

Transition State 4	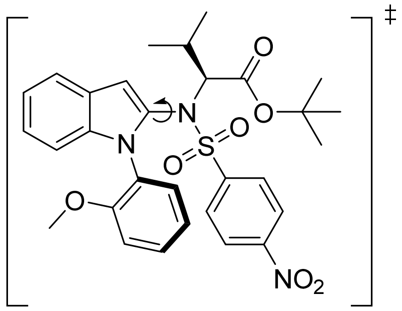

	Geom.opt.		Single-point		
level	M06-2X/def2SVP		M06-2X/def2TZVP		
energy	Thermal correction to Gibbs Free Energy =0.44876hartree		E =-2134.0346 hartree		
cartesian coordinates of stationary point structure [Å]	C	0.74234400	-4.46895200	-6.11823600	
	C	0.01362200	-5.33256700	-5.23335900	
	C	0.42372500	-6.65133400	-4.97462300	
	C	1.59621700	-7.08102800	-5.58138100	
	C	2.33978700	-6.23226800	-6.43643600	
	C	1.92553800	-4.93585800	-6.71293400	
	C	0.01592500	-3.22863000	-6.20317300	
	C	-1.11277900	-3.31102800	-5.37848100	
	H	-0.15316800	-7.29930300	-4.32058000	
	H	1.95691400	-8.09294200	-5.39980700	
	H	3.25481700	-6.61440700	-6.88760000	
	H	2.49497900	-4.28993300	-7.37709600	
	H	0.35857000	-2.39809000	-6.78792800	
	N	-1.13136400	-4.60766700	-4.74792400	
	C	-2.25846000	-5.40442300	-4.31599100	
	C	-2.55467100	-5.65838200	-2.94161200	
	C	-3.14024600	-5.89749700	-5.29999400	
	C	-3.72450100	-6.37035300	-2.59898600	
	C	-4.27851200	-6.61989800	-4.94614900	
	C	-4.57105900	-6.84423200	-3.59808400	
	H	-3.96908400	-6.56414400	-1.55647200	
	H	-4.94194000	-7.00014000	-5.72187400	
	H	-5.46968200	-7.40106500	-3.32114000	
	N	-1.99671100	-2.21432300	-5.14161100	
	C	-2.93130000	-2.19217900	-3.99086300	
	H	-3.44046500	-1.16260700	-3.99088700	
	C	-4.11269700	-3.14669400	-4.03212600	
	H	-5.06022500	-2.59097600	-4.01969000	
	H	-4.12890300	-3.74088200	-4.95388200	
	H	-4.12750800	-3.83462900	-3.17352700	
	C	-2.19181300	-2.17487600	-2.62015000	
	O	-2.65160100	-2.53126800	-1.55415700	
	O	-0.99204500	-1.55314700	-2.78268500	
	C	-0.13900400	-1.02133800	-1.67003100	
	C	-0.96501800	-0.05907900	-0.82142700	
	C	0.42898800	-2.17242500	-0.84882800	
	C	0.96739700	-0.31944500	-2.45742800	
	H	-1.44168900	0.72344900	-1.41920300	

	H	-1.75805600	-0.57925700	-0.26768000
	H	-0.33050400	0.44885100	-0.08343900
	H	1.00915700	-2.86914500	-1.46855600
	H	1.10966000	-1.80161300	-0.07178900
	H	-0.36285100	-2.73575000	-0.34627700
	H	1.72458600	0.09781700	-1.78419700
	H	1.46943900	-1.01523500	-3.14397000
	H	0.58649300	0.50245200	-3.07845500
	S	-1.50395400	-0.68374100	-5.87322800
	O	-0.18481500	-0.32887200	-5.41461000
	O	-1.70055600	-0.88392900	-7.29403100
	C	-2.65522200	0.49512100	-5.33566300
	C	-2.34066000	1.35903800	-4.28285900
	C	-3.85881400	0.63453100	-6.03817800
	C	-3.24416400	2.35458300	-3.89514500
	H	-1.37697300	1.26363000	-3.75636700
	C	-4.77702900	1.62356500	-5.66470500
	H	-4.09111100	-0.02460100	-6.88646700
	C	-4.45759000	2.46956700	-4.59131800
	H	-3.00900400	3.03727700	-3.06777800
	H	-5.72640400	1.74019600	-6.20361700
	N	-5.42864300	3.52380800	-4.18514500
	O	-6.46539200	3.63310400	-4.82979500
	O	-5.14351100	4.23115500	-3.22505700
	H	-2.91750700	-5.71081700	-6.35518200
	O	-1.63575200	-5.21682000	-2.01770400
	C	-1.98947300	-5.44664800	-0.61673200
	H	-2.16673000	-6.51326400	-0.42084800
	H	-1.09779000	-5.09370000	-0.07572800
	H	-2.87229200	-4.83674800	-0.36601200
